# Fully Synthetic, Biomimicking Polysulfates With Tunable Anticoagulant and Endothelial Cell–Selective Bioactivity

**DOI:** 10.1002/mabi.70201

**Published:** 2026-06-07

**Authors:** Andrea Cosimi, Roxana Pollehn, Andrea De Martino, Scarlett Manzke, Yannic Kerkhoff, Philip Nickl, Theresa Lohmann, Eriselda Keshi, Cornelia Lee‐Thedieck, Marie Weinhart

**Affiliations:** ^1^ Institute of Chemistry and Biochemistry – Organic Chemistry Freie Universität Berlin Berlin Germany; ^2^ Institute of Physical Chemistry and Electrochemistry Leibniz Universität Hannover Hannover Germany; ^3^ IT & Data Services Zuse Institute Berlin Berlin Germany; ^4^ Department of Surgery Campus Charité Mitte | Campus Virchow‐Klinikum Experimental Surgery Charité – Universitätsmedizin Berlin Corporate Member of Freie Universität Berlin Humboldt‐Universität Zu Berlin Berlin Germany; ^5^ Berlin Institute of Health (BIH) Berlin Germany; ^6^ Institute of Cell Biology and Biophysics Leibniz Universität Hannover Hannover Germany

**Keywords:** GAG‐mimetic, growth factor sequestration, in vitro reendothelialization, polyelectrolyte brushes, sulfated PHEMA

## Abstract

We present a versatile method for fabricating glycosaminoglycan (GAG)‐inspired polyelectrolyte brush coatings from fully synthetic sulfated PHEMA block copolymers. Using a bioinert backbone enables evaluation of sulfation effects independently of natural GAGs’ carbohydrate backbone. A degree of sulfation above 70% imparted anticoagulant activity, extending plasma coagulation times beyond 500 s at 0.1 mg mL^−^^1^. Controlled self‐assembly enabled fabrication and photoimmobilization of uniform, nanometer‐thin brushes on polystyrene substrates. The polysulfate brushes exhibited molecular weight‐dependent properties: under serum‐free conditions, endothelial cells (HUVECs) selectively proliferated on longer **P2**‐OSO_3_‐BP (65 kDa) compared to shorter **P1**‐OSO_3_‐BP (15 kDa) brushes, while smooth muscle cells (SMCs) remained quiescent. Despite comparable VEGF and bFGF surface densities (0.5 ng cm^−2^), **P2**‐OSO_3_‐BP coatings better preserved VEGF bioactivity, likely due to higher chain flexibility. In co‐culture under serum conditions (5%), HUVEC/SMC ratios remained near unity with persistent colocalization, indicating restrained SMC overgrowth and stabilized vascular co‐culture relevant to preventing neointimal hyperplasia. These findings highlight synthetic polysulfate brush coatings as a platform for studying sulfation‐driven growth factor interactions and vascular cell competition at biomaterial interfaces, promoting reendothelialization in vitro. The system therefore represents a functional mimetic of GAGs, reproducing key electrostatic features while avoiding the structural complexity of native polysaccharides.

## Graphical abstract

Sulfated PHEMA‐based polyelectrolyte brushes are introduced as a reductionist platform to probe glycosaminoglycan‐inspired bioactivity. High charge density governs growth factor interactions and is associated with distinct vascular cell responses, enabling balanced endothelial and smooth muscle cell co‐culture. These fully synthetic coatings decouple structural complexity from function, offering tunable and cell‐instructive interfaces for blood‐contacting biomaterials and implant coatings.

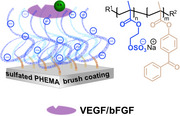

## 1 Introduction

Glycosaminoglycans (GAGs), such as heparan sulfate or dermatan sulfate, are linear, highly charged polysaccharides embedded within the extracellular matrix (ECM) and glycocalyx, where they play essential roles in modulating cell adhesion, signaling, inflammation, and hemostasis [1]. Their bioactivity is largely driven by the spatial arrangement and density of anionic sulfate and carboxylate groups, which mediate electrostatic interactions with a broad spectrum of proteins and cell surface receptors [2, 3]. The complex and heterogeneous sulfation patterns govern specific protein binding and stabilization. Particularly, GAG‐growth factor interactions are critical for processes such as angiogenesis and tissue regeneration [4] by facilitating receptor‐mediated cell signaling via, e.g., basic fibroblast (bFGF) and vascular endothelial growth factor A (VEGF‐A) [5, 6]. However, biological variability and production‐related limitations pose significant challenges and costs for their safe, reproducible, and scalable use in biomedical research and clinical applications [7].

To address these limitations, synthetic GAG mimetics—particularly sulfated polymers—have emerged as promising alternatives [8, 9]. These materials aim to replicate the multivalent anionic features of GAGs in a structurally defined and chemically stable manner. While the majority of research on synthetic GAG mimetics has focused on saccharide‐based polymers and on the anticoagulant and antiviral activities associated with their specific sulfation patterns and charge distributions [9, 10, 11, 12], sulfated polymers also hold considerable potential for modulating cell‐material interactions [13]. By mimicking heparan sulfate´s ability to bind to growth factors and present them to cell receptors, these polymers enhance adhesion, proliferation, and differentiation of different cell types in vitro [14, 15, 16]. Importantly, this cell‐instructive capacity makes GAG mimetics directly relevant for blood‐contacting materials, where controlled interactions between the biomaterial surface and vascular cells dictate long‐term function. Blood‐contacting materials, such as vascular grafts, stents, and catheters, are exposed directly to blood flow and must therefore mimic native vessel interfaces to avoid thrombosis, inflammation, or graft failure [17, 18]. Rapid reendothelialization—the process by which endothelial (progenitor) cells form a confluent, functional lining over a biomaterial surface—is crucial to restore barrier function, reduce platelet adhesion, suppress coagulation, and prevent excessive smooth muscle cells (SMCs) proliferation and migration, which otherwise leads to neointimal hyperplasia and graft failure [19]. The ideal outcome is therefore a coating that reliably promotes the rapid formation of a stable, functional endothelial monolayer within days to weeks, resists thrombosis under physiological conditions, and suppresses SMCs’ overgrowth [20, 21, 22]. Among various formats [23, 24, 25], surface‐grafted, GAG‐mimicking polyelectrolyte brushes offer a versatile strategy for reproducibly engineering cell‐instructive and safe blood‐contacting interfaces, emulating aspects of ECM topology and biofunction to control the biological response [26, 27].

Building on this concept, we hypothesize that introducing high‐density negative charges to a bioinert, chemically simple polymer backbone could impart GAG‐like features to the system both in solution and on surfaces. As a model system, we selected poly(2‐hydroxyethyl methacrylate) (PHEMA)—a biocompatible and FDA‐approved polymer– due to its known bioinertness and the availability of multiple hydroxyl groups for chemical conversion into sulfate groups. Since most PHEMA‐coated surfaces generally do not support protein‐surface interactions or the attachment of anchorage‐dependent mammalian cells unless modified with cell adhesive peptide motifs like the arginine, glycine, aspartic acid (RGD)‐tripeptide [28, 29], the contribution of introduced negative charges can be systematically evaluated.

To test our hypothesis, we first synthesized HEMA‐based homo‐ and block copolymers via reversible addition‐fragmentation chain‐transfer (RAFT) polymerization. These polymers were then sulfated to varying degrees to systematically modulate their charge density. This allowed us to evaluate their GAG‐inspired bioactivity both in solution—via blood plasma coagulation assays—and on surfaces—via human primary cell adhesion and proliferation studies in mono‐ and co‐culture settings. By comparing the cell behavior on fully sulfated and non‐sulfated polymer coatings, we could assess the contribution of multivalent anionic charge motifs relative to the simplified polymer backbone, independent of the structural complexity of native GAG polysaccharides. Importantly, our approach does not aim to replicate the full structural complexity of natural GAGs, but rather to decouple and investigate sulfate‐mediated electrostatic interactions within a simplified, well‐defined system. Such a bottom‐up, modular strategy provides a reductionist yet tunable platform for dissecting how charges and polymer architecture—including chain length and grafting density—govern biological responses. In turn, these insights provide critical information for the functionally optimized design of fully synthetic GAG‐inspired materials, which reproduce selected physicochemical features (e.g., high charge density) rather than the full structural and biochemical complexity of native polysaccharides, for tailored biomedical applications, including anticoagulant and cell‐instructive surfaces such as bioactive implant coatings.

## 2 Results and Discussion

**SCHEME 1:**
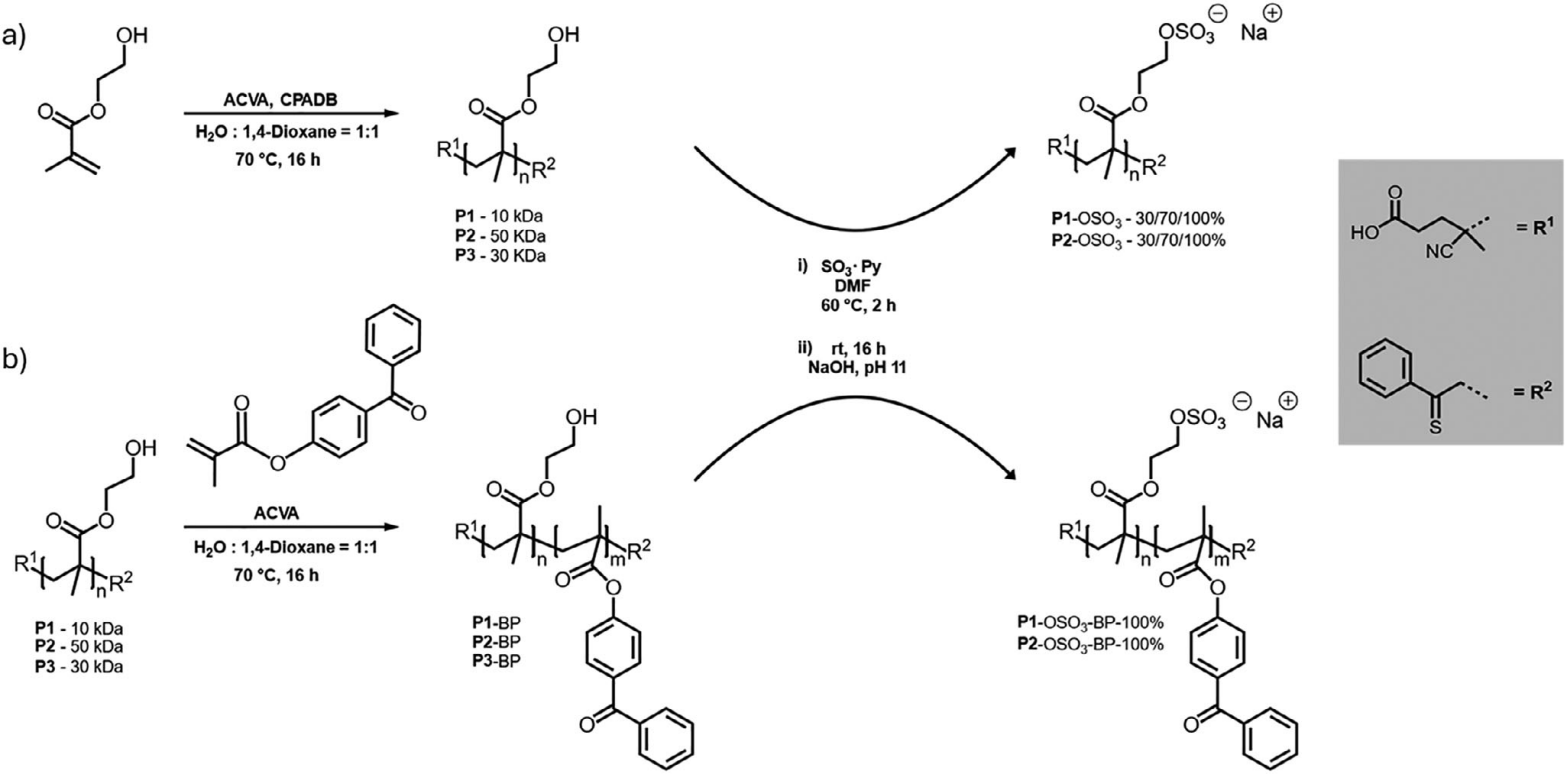
Synthesis and postfunctionalization of PHEMA‐based polymers. (a) RAFT polymerization of HEMA with (b) sequential addition of a benzophenone (BP)‐based comonomer using 4,4´‐azobis(4‐cyanovaleric acid) (ACVA) as initiator and 4‐cyano‐4‐(phenylcarbonothioylthio)pentanoic acid (CPADB) as chain transfer agent to prepare PHEMA homopolymers **P1‐3** and block copolymers **P1‐3**‐BP comprising a short, photo‐reactive BP anchor block for polymer self‐assembly and covalent surface immobilization. Postmodification of the hydroxyl groups via sulfation yielded **P1‐2**‐OSO_3_ with targeted degrees of sulfation (30, 70, 100%) and the respective quantitatively sulfated block copolymers **P1‐2**‐OSO_3_‐BP.

**FIGURE 1:**
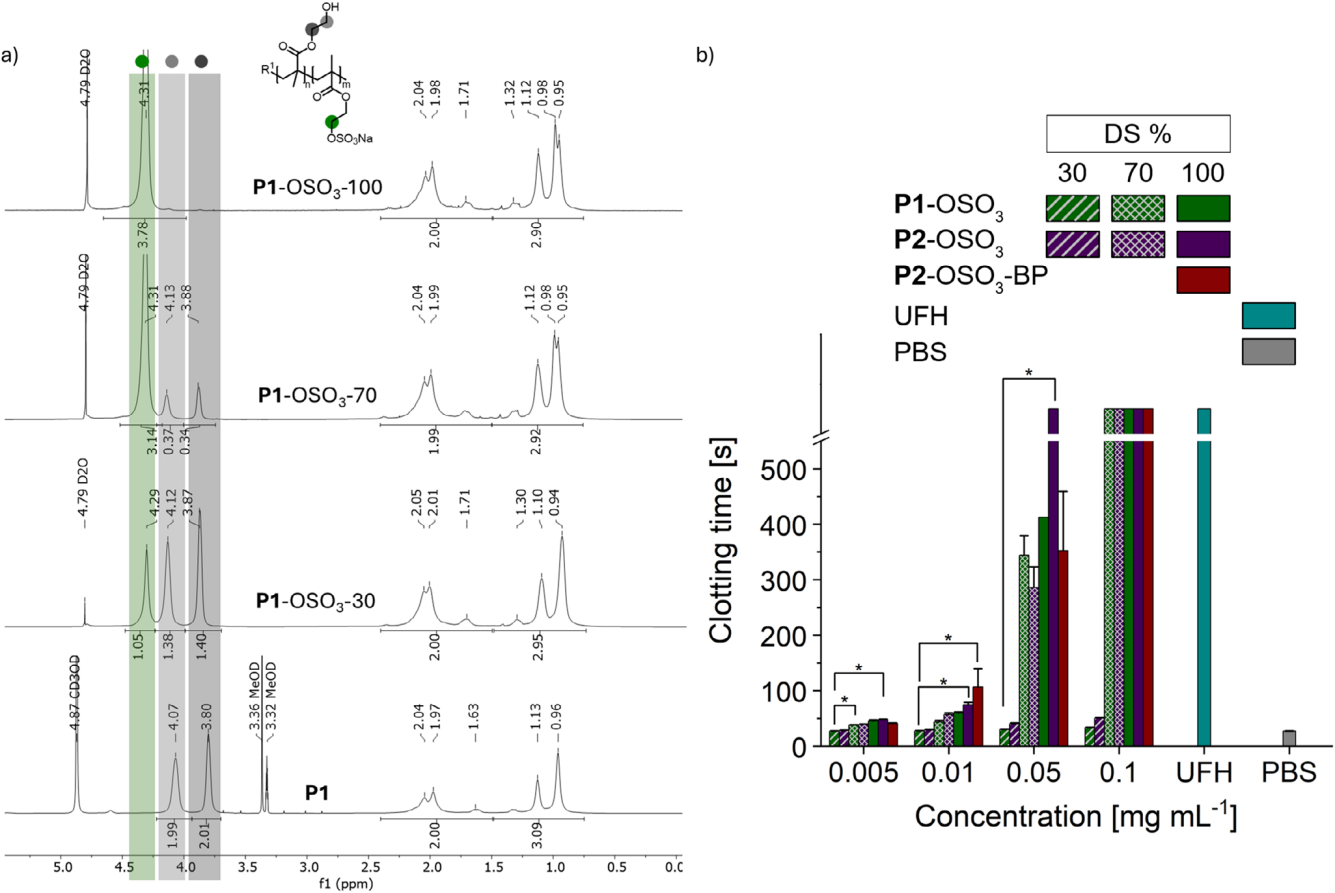
(a) Representative ^1^H NMR spectra of **P1**‐OSO_3_ with different DS (30, 70, 100%) in D_2_O compared to the unsulfated precursor **P1** recorded in CD_3_OD. (b) Concentration‐dependent anticoagulant profiles of **P1**‐OSO_3_, **P2‐**OSO_3_, and **P2‐**OSO_3_‐BP with varying DS based on a PHEMA backbone of 15 and 65 kDa as determined in an aPTT assay, along with unfractionated heparin (UFH) and PBS as controls. Statistical significance was tested with a non‐parametric Kruskal‐Wallis ANOVA test. (^*^, *p* < 0.05) (n = 3).

To design GAG‐inspired PHEMA‐based polymer coatings, first linear PHEMA homopolymers with a targeted molecular weight of 10, 30, and 50 kDa (**P1‐3**) were synthesized via RAFT polymerization, adapted from Tischer et al. [30]. Molecular weight analysis by ^1^H NMR spectroscopy (Figures S2–S4), using the signals of 4‐cyano‐4‐(phenylcarbonothioylthio)pentanoic acid (CPADB) chain‐end fragments as references, indicated slightly higher values than the targeted ones (Table 1). In contrast, gel permeation chromatography (GPC) analysis in dimethyl formamide (DMF) using polymethyl methacrylate (PMMA) standards yielded higher apparent molecular weights, with values approximately twice the theoretical targets. This discrepancy is described in the literature for methacrylate‐based polymers in DMF and arises from differences in hydrodynamic volume and polymer‐solvent interactions between PHEMA and PMMA standards [31, 32]. However, reasonable dispersities between *Ð* = 1.22–1.37 were detected, indicating a controlled polymerization essential for block copolymer formation. In a second step, the hydroxyl groups of **P1‐2** were sulfated using 1.1 equivalents of sulfur trioxide‐pyridine complex (SO_3_·py) per targeted OH group in dry DMF to yield polysulfates **P1‐**OSO_3_ and **P2‐**OSO_3_ (Scheme 1) with varying degrees of sulfation (DS), aiming at 30%, 70%, and 100% (Table 1). The targeted DS corresponds well to the experimentally determined values based on elemental analysis (Table S1) and peak integration of the newly appearing ^1^H NMR signal at around 4.3 ppm (Figure 1a). This signal corresponds to the four methylene protons in the side chains of HEMA units upon hydroxyl group sulfation, which are originally located at 3.86 and 4.12 ppm in the non‐sulfated PHEMA precursor. Although both methods show good agreement, the DS calculated from ^1^H nuclear magnetic resonance (NMR) peak integration is consistently higher (15 – 40%) than the values obtained by elemental analysis. This deviation is likely due to the hygroscopic nature of the lyophilized polymers, which can lead to an underestimation of the sulfur content during elemental analysis. In contrast, the NMR‐based determination remains reliable, as the signals of interest are well resolved and do not suffer from peak overlap. Aqueous GPC analysis using polystyrene sulfonate (PSS) standards tends to underestimate the molecular weight, depending on the DS. Thus, the molecular weights calculated from ^1^H NMR spectra are considered most reliable. These sulfated homopolymers provide a simplified model to explore their presumed GAG‐like properties in solution, arising from statistically distributed charges along a chemically defined PHEMA backbone.

**TABLE 1:** Molecular weight characteristics and comonomer composition via the repeating units (r.u.) of PHEMA‐based homo‐ and block copolymers synthesized via RAFT, followed by optional postmodification via sulfation targeting a DS of 30%, 70%, and 100%.

Polymer	*M_n_* ^theo^ [kDa]	*M_n_* ^NMR^ [kDa]	*M_n_* ^GPC^ [kDa]	*Đ* ^GPC^	r.u.^a^ (‐OH)	r.u.^b^ (‐OSO_3_Na)	r.u.^a^ (BP)	DS^NMR^ ^b^ [%]	DS^EA^ ^e^ [%]
**P1**	10	15^a^	31^c^	1.41	119	n.a.	n.a.	n.a.	n.a.
**P2**	50	65^a^	128^c^	1.31	496	n.a.	n.a.	n.a.	n.a.
**P3**	30	36^a^	55^c^	1.22	280	n.a.	n.a.	n.a.	n.a.
**P1‐**OSO_3_‐30	13	21^b^	19^d^	1.83	68	51	n.a.	43	31
**P1‐**OSO_3_‐70	16	27^b^	12^d^	1.75	12	107	n.a.	90	76
**P1‐**OSO_3_‐100	18	28^b^	16^d^	1.6	0	119	n.a.	100	87
**P2‐**OSO_3_‐30	60	87^b^	53^d^	1.46	273	223	n.a.	45	32
**P2‐**OSO_3_‐70	82	106^b^	60^d^	1.48	90	406	n.a.	82	66
**P2‐**OSO_3_‐100	90	115^b^	70^d^	1.41	0	496	n.a.	100	87
**P1**‐BP	12	17^a^	35^c^	1.29	119	n.a.	5	n.a.	n.a.
**P2‐**BP	52	67^a^	142^c^	1.39	496	n.a.	7	n.a.	n.a.
**P3‐**BP	32	37^a^	57^c^	1.25	280	n.a.	5	n.a.	n.a.
**P1‐**OSO_3_‐BP	30	29^b^	18^d^	1.53	0	119	5	100	n.d.
**P2‐**OSO_3_‐BP	105	117^b^	108^d^	1.98	0	496	7	100	95

^a^ as determined by ^1^H NMR spectroscopy in CD_3_OD.

^b^ as determined by ^1^H NMR spectroscopy in D_2_O.

^c^ as determined by GPC measurements in 0.01 M LiBr in DMF applying PMMA standards.

^d^ as determined by GPC measurements in 0.08 M Na_2_HPO_4_ in H_2_O applying PSS standards.

^e^ as determined by elemental analysis (EA). n.a. = not applicable, n.d. = not determined.

Alternatively, **P1‐3** homopolymers were converted in situ into PHEMA‐based block copolymers **P1‐3**‐BP by sequential RAFT polymerization to introduce an oligomeric benzophenone (BP)‐comprising block (Scheme 1b). The presence of additional aromatic signals in the ^1^H NMR spectra of **P1‐3**‐BP confirmed successful BP‐block incorporation (Figures S5, S7, and S9). Aromatic peak integration revealed, on average, 5–7 BP units per anchor block (Table 1) according to the calculation outlined in the Supporting Information (SI). GPC analysis of the block copolymers showed a slight increase in molecular weight and dispersity compared to the homopolymers, further supporting successful sequential polymerization. GPC traces of both the homopolymers and the corresponding copolymers (Figures S6, S8, and S10) show predominantly unimodal weight distributions, with a clear shift toward higher molecular weights upon chain extension, while no low‐molecular‐weight shoulders are observed. These block copolymers’ hydroxyl groups were then quantitatively sulfated, as determined by ^1^H NMR spectroscopy (Figures S11, and S13). Notably, the decrease of the integral associated with the MABP region observed for the resulting polymers **P1‐2**‐OSO_3_‐BP in D_2_O compared to CD_3_OD is attributed to the restricted solubility of the MABP block. In aqueous solution, the amphiphilic nature of these block copolymers results in micelle formation, in which the MABP segments are located in the core and are therefore difficult to detect by ^1^H NMR [33]. Consequently, the aromatic peak integrals are attenuation due to restricted chain mobility and cannot be used for quantitative analysis. Consistently, aqueous GPC analysis using PSS standards showed unimodal distribution (Figures S12–S14) and yielded molecular weights that are lower (**P1**‐OSO_3_‐BP) or comparable (**P2**‐OSO_3_‐BP) to the theoretical values, in agreement with the sulfated homopolymers (Table 1). The resulting polymers **P1‐2**‐OSO_3_‐BP enable the formation of polyelectrolyte brush coatings through self‐assembly and subsequent photoimmobilization on surfaces.

First, we evaluated the anticoagulant activity of sulfated PHEMA in solution via an activated partial thromboplastin time (aPTT) assay using human blood plasma (Figure 1b), as a model to assess sulfate‐mediated electrostatic interactions with proteins while reducing the structural complexity associated with native GAGs. The concentration‐dependent anticoagulant profiles of **P1**‐OSO_3_ and **P2**‐OSO_3_ based on a 15 and 65 kDa PHEMA backbone with varying DS (30%, 70%, and 100%) were determined. Unfractionated heparin (UFH; 201 USP mg^−1^) and phosphate‐buffered saline (PBS) served as positive and negative controls, respectively. To investigate the impact of the BP‐anchor block on blood coagulation, the corresponding profile of block copolymer **P2**‐OSO_3_‐BP based on a 65 kDa PHEMA backbone with 100% sulfation was determined as well. The aPTT assay evaluates the intrinsic pathway of blood coagulation [34], in which prolonged plasma clotting times exceeding 25–35 s typically indicate an anticoagulated state. Expectedly, increasing anticoagulant activity was observed with increasing backbone molecular weight and increasing DS [13, 35, 36], which became most pronounced at a concentration of 0.05 mg mL^−1^ and above. Similar to other synthetic polysulfates [36, 37], the coagulation time increased with polymer concentration. At 0.1 mg mL^−1^, the coagulation times of all sulfated PHEMA polymers with a minimum DS of 70% exceeded 500 s, which was the maximum time of observation (Figure 1b). For UFH, this anticoagulant effect was already reached at 10 times lower concentration, which corresponded to 2 USP mL^−1^ for this specific batch of heparin. Importantly, the short BP‐based anchor block in **P2**‐OSO_3_‐BP induced no significantly different coagulation profile when compared to **P2**‐OSO_3_ with a comparable backbone molecular weight and DS. Overall, these results confirm the anticoagulant activity of highly sulfated PHEMA polymers (DS ≥ 70%), underscoring their potential as promising candidates for the preparation of blood‐contacting coatings. While structurally less complex than native GAGs, these systems reproduce the high density of sulfate groups that govern electrostatic interactions with coagulation factors.

**FIGURE 2:**
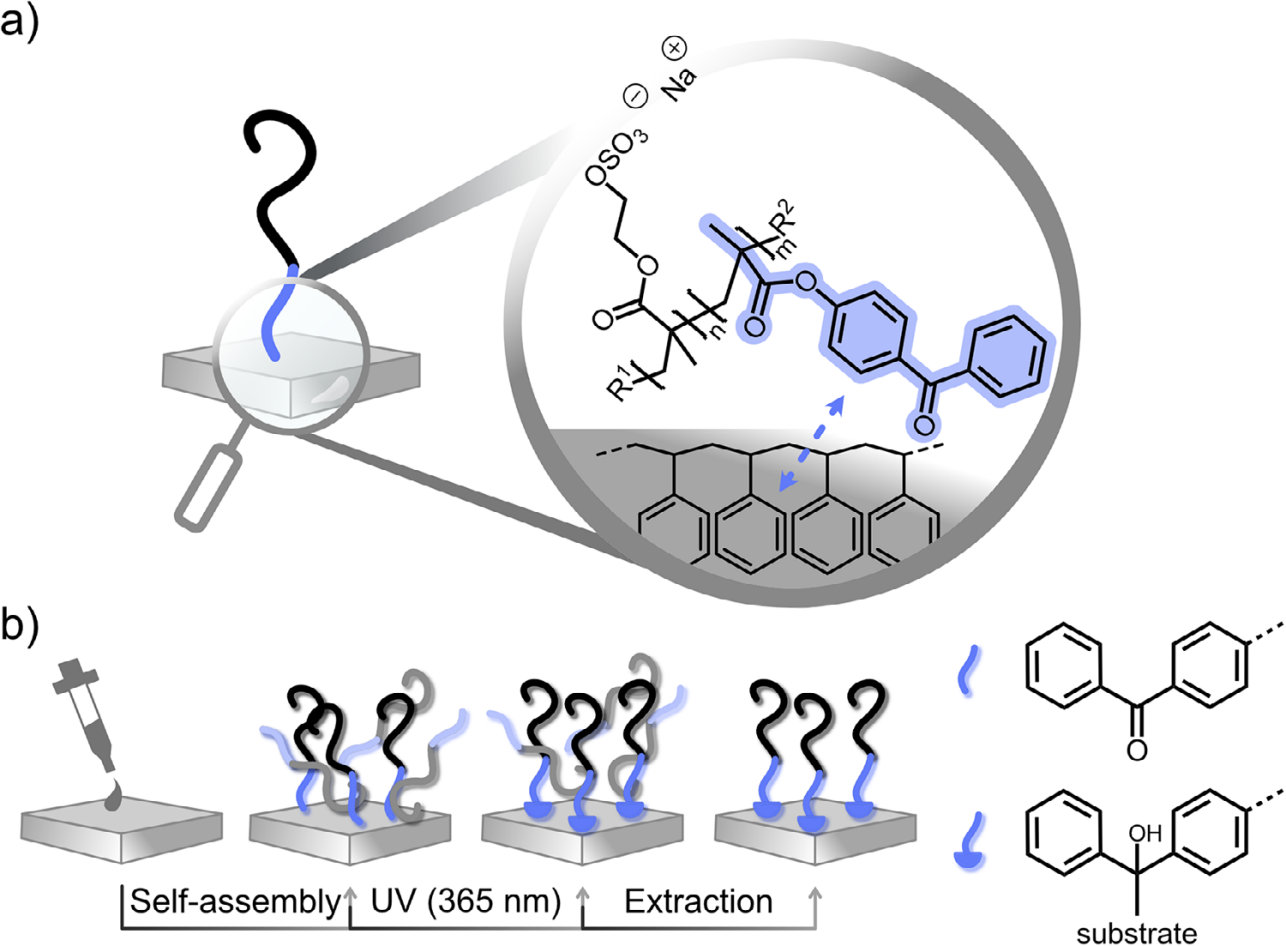
Schematic representation of the self‐assembling of sulfated PHEMA block copolymers with (a) chemical structures indicating the expected interactions with a PS substrate and (b) illustration of the coating procedure via a “grafting‐to” approach.

Previous studies [38] have shown that heparin‐like coatings based on saccharide‐ and sulfonated monomers on polyurethane substrates support the adhesion and proliferation of HUVECs for three days. Additional anticoagulant activity made these coatings promising candidates for blood‐contacting materials that promote reendothelialization, critically needed for, e.g., stents. To investigate the cellular response of primary cells cultured on coatings prepared from sulfated PHEMA of varying chain length, quantitative sulfation was envisioned to ensure the highest anticoagulant potential, together with a non‐sulfated control. BP‐comprising block copolymers **P1**‐OSO_3_‐BP and **P2**‐OSO_3_‐BP with a PHEMA backbone of 15 and 65 kDa, and **P3**‐BP with an intermediate PHEMA backbone molecular weight of 36 kDa were employed to establish brush‐like coatings on polystyrene (PS) substrates (Figure 2). While **P3**‐BP does not represent a strictly matched molecular weight control, it serves as a non‐sulfated reference to assess baseline cell and protein interactions in the absence of sulfation. BP units in copolymers are well‐established photoactive anchors for functionalizing plastic substrates with a wide range of polymers, as they undergo UV light‐induced C, H‐insertion into the substrate's hydrocarbon groups upon irradiation at ∼ 365 nm [39], ensuring stable covalent polymer attachment. When incorporated as short terminal blocks in copolymers, the BP units can drive the self‐assembly of the block copolymer through hydrophobic interactions with the substrate, forming brush‐like coatings with tunable grafting density via the polymer concentration [40, 41, 42]. For example, poly(glycidyl ether) (PGE)‐based block copolymers with short BP‐based anchor blocks have demonstrated efficient self‐assembly on both hard plastics [42] and soft hydrogels [43]. The substrate affinity of the BP‐anchor block critically depends on the coating conditions, such as the choice of a selective solvent: it should solvate the functional polymer block effectively while minimizing solubility of the BP block to promote hydrophobic interactions with the solid surface. In addition, the substrate affinity of the anchor block can be tuned through the flexibility and chemical nature of the linker connecting the BP units with the polymer backbone [42, 44].

To establish a reliable coating protocol for PS with charged BP‐comprising block copolymers, aiming for maximum thickness and surface coverage of the resulting polyelectrolyte brushes, it is essential to first identify the proper selective solvent conditions. Prior work using linear polyglycerol sulfate (lPGS) block copolymers with a similar BP anchor but enhanced hydrophilicity demonstrated the formation of localized aggregates of around 8 nm in height, covering only 50% of the PS surface [45]. These aggregates are attributed to the self‐assembly of the block copolymers into micellar structures in aqueous salt solution, which deposit as such on the surface and result in a patchy, inhomogeneous coating with incomplete surface coverage [45]. The size of the micelles strongly depends on the ionic strength of the salt solution and the chaotropic or kosmotropic nature of the salt. Therefore, dynamic light scattering (DLS) was conducted to evaluate the micellar aggregation behavior of the sulfated PHEMA‐based copolymers **P1**‐OSO_3_‐BP and **P2**‐OSO_3_‐BP in water and NaCl solutions (0.1–1 m) before (Figure S15a) and after filtration (0.2 µm) (Figure S15b). To disfavor micelle formation, the polymer concentration was intentionally maintained low (∼ 10^−5^ M). For **P1**‐OSO_3_‐BP, colloidal sizes between 5 and 10 nm were detected across all conditions, indicating solubilized single chains in salt‐free conditions. In contrast, **P2**‐OSO_3_‐BP formed ∼ 800 nm aggregates in water as similarly observed with lPGS block copolymers (∼ 250 nm) at a similar concentration (0.1 mg mL^−1^, 2.3 µM) with comparable *M*_n_ and DS [45]. **P2**‐OSO_3_‐BP aggregates could be attributed to micelle formation due to the BP block, as control experiments with **P2**‐OSO_3_ lacking the BP block revealed sizes of respectively 10 and 6 nm in water and in 0.8 M NaCl (data not shown). For lPGS‐based micelles, the addition of salt generally reduced the hydrodynamic diameter to around 90 nm due to charge screening [45], which induces the collapse and contraction of the polyelectrolyte‐based micellar shell. Similarly, the presence of only 0.1 M NaCl reduced the size of **P2**‐OSO_3_‐BP micelles to approximately 30 nm with only minor variations up to 1 M NaCl (Figure S15a). Additional application of shear stress during filtration further reduced the colloidal size of **P2**‐OSO_3_‐BP to 20–25 nm (Figure S15b), supporting our interpretation that the higher‐molecular‐weight block copolymers form shear‐sensitive micelles. Overall, the presence of ≥ 0.1 M NaCl, combined with additional filtration of the aqueous solution at room temperature (RT), proved essential to substantially reduce micellation and favor predominantly dispersed block copolymer polyelectrolyte chains at low concentrations.

Establishing selective solvent conditions for **P3**‐BP (*M*_n_ = 57 kDa) to prepare a non‐sulfated control coating was more challenging and required a mixture of water‐ethanol at 1:9 (v/v%) ratio. At this specific ratio, the differential solubility of the HEMA and BP blocks is exploited to enhance BP‐substrate interactions, similarly to the optimized coating protocol with charge‐neutral PGE block copolymers [42]. Water preferentially solubilizes the HEMA block, whereas ethanol enhances the solubilization of the BP block and promotes its orientation toward the substrate. Again, DLS data in aqueous ethanol helped to optimize the composition (data not shown) and temperature of the selective solvent for efficient coating conditions, based on the aggregation behavior of **P3**‐BP (Figure S15c). Filtration of **P3**‐BP in aqueous ethanol (10 v/v%) at 20 °C had only a minor effect on aggregate size in the selective solvent, reducing it from 20 to 12 nm at a concentration of ∼ 7 µM (250 µg mL^−1^). However, increasing the temperature to 40 °C partially disrupted the aggregates. The resulting bimodal distribution at 40 °C after filtration indicates the presence of both individual polymer chains (∼ 6 nm) and loosely associated aggregates (∼13 nm) (Figure S15c). To further verify that self‐assembly from the selective solvent on the PS surface is primarily governed by the BP‐substrate interactions rather than the PHEMA block, we used quartz crystal microbalance with dissipation (QCM‐D). The dynamic adsorption of **P3** (without BP) and **P3**‐BP on PS‐coated QCM‐D gold sensors under diluted conditions (250 µg mL^−1^) in both selective (aq. EtOH) and non‐selective (EtOH) solvents was monitored at room temperature. As expected, both polymers **P3** and **P3**‐BP exhibited negligible surface adsorption in EtOH, as indicated by the minimal frequency shifts (Δ*f*) observed upon polymer exposure. In contrast, in the selective solvent, **P3**‐BP exhibited a significantly larger frequency shift Δ*f* than **P3**, corresponding to an adsorbed areal mass of 380 ± 20 ng cm^−2^ (Figure S16), thereby confirming BP‐mediated surface interactions. Dissipation shifts (Δ*D*) during polymer exposure further revealed that the thin films formed from **P3** adsorption were loosely bound and disordered regardless of the solvent, as indicated by the moderate Δ*D* shifts (4.5 × 10^−6^) in both conditions (Figure S16b). Substantially lower Δ*D* shifts (0.5 × 10^−6^) during self‐assembly of **P3**‐BP from selective solvents are indicative of a more structured and compact layer characteristic for oriented polymer brushes.

**FIGURE 3:**
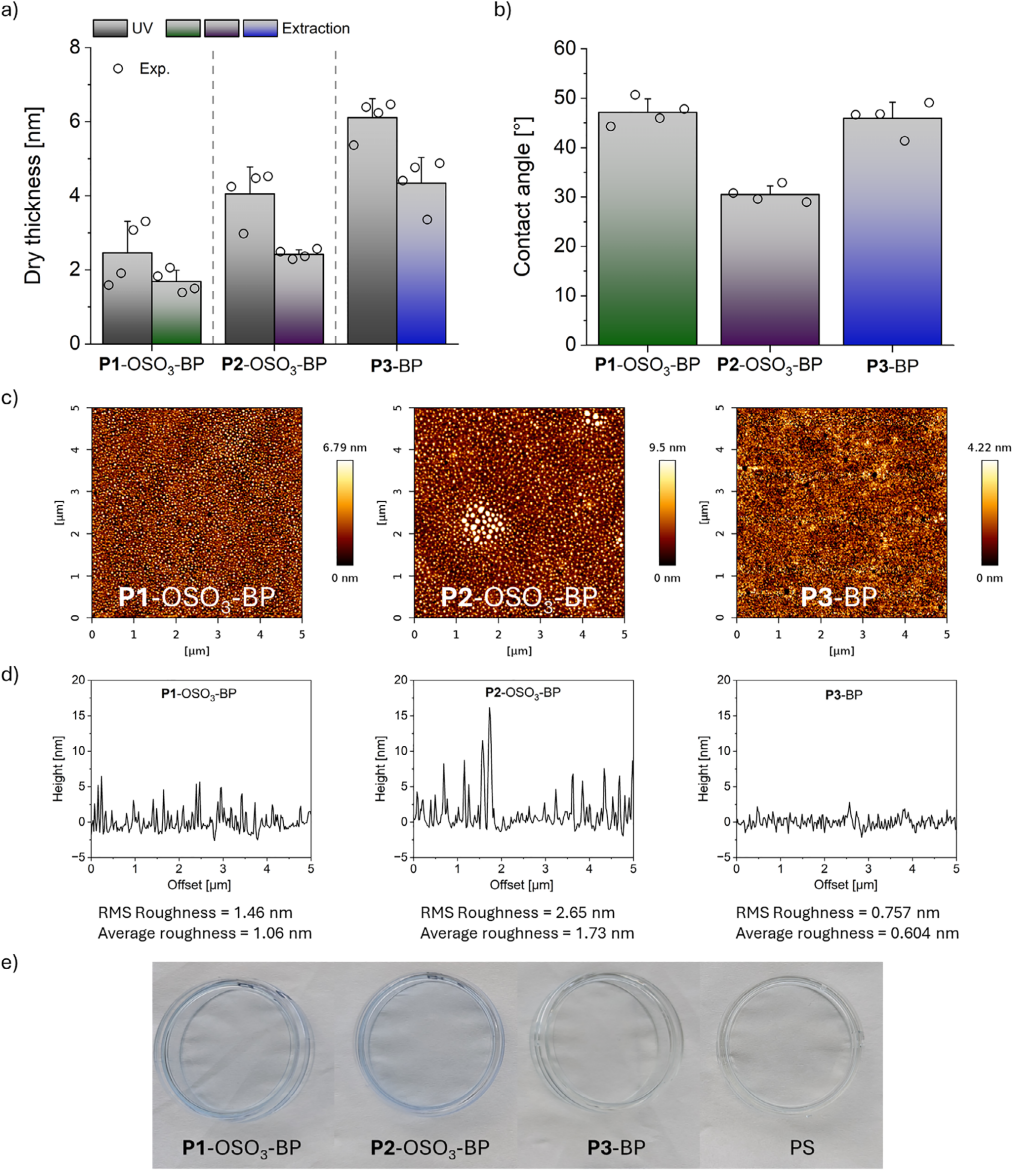
(a) Dry layer thickness as measured via SE after UV immobilization (grey bars) and water extraction (colored bars), and (b) CA measured at RT (21°C) of **P1**‐OSO_3_‐BP (green), **P2**‐OSO_3_‐BP (purple), and **P3**‐BP (blue) brushes on PS‐coated silicon wafer substrates. Empty symbols represent the experimental data points. Error bars represent the standard deviation. (n = 4) (c) Representative AFM topography and (d) the respective cross‐section of **P1**‐OSO_3_‐BP, **P2**‐OSO_3_‐BP, and **P3**‐BP. The cross sections are averaged over the whole scanned area. (e) Representative photographs of methylene blue‐stained Petri dishes coated with **P1**‐OSO_3_‐BP, **P2**‐OSO_3_‐BP, **P3**‐BP control, and native PS control after 24 h incubation in 1 mM dye solution at RT, followed by extensive washing with Milli‐Q water.

For coating and subsequent straightforward surface characterization, PS‐coated silicon wafers were statically incubated with the block copolymer solution at concentrations in the µM range under optimized conditions (solvent composition, ionic strength, and temperature) for 90 min to ensure singularized polymer chains. After rinsing and drying, the substrates were UV‐irradiated (365 nm) and exhaustively extracted with ethanol (**P3**‐BP) or water (**P1**‐ and **P2**‐OSO_3_‐BP) to remove non‐covalently bound material, as illustrated in Figure 2b. The resulting coatings were analyzed by spectroscopic ellipsometry (SE), static water contact angle (CA), and atomic force microscopy (AFM) to determine dry film thickness, wettability, and surface morphology. Although DLS data indicated singularized chains at NaCl concentrations ≥ 0.1 M, polyelectrolyte brush coatings were prepared under varying ionic strengths to identify the optimal salt concentration, based on the resulting dry thickness after 16 min of UV exposure and subsequent exhaustive extraction (Figure S15d, e). Interestingly, no self‐assembly—not even in patches—occurred from pure water, where the block copolymers formed micellar aggregates. From salt‐containing solutions, dry thicknesses between 1.4–2.0 nm for **P1**‐OSO_3_‐BP (Figure S15d) and 1.3–2.4 nm for **P2**‐OSO_3_‐BP (Figure S15e) were detected, with the highest thicknesses achieved from 0.8 M NaCl solutions. Therefore, subsequent polyelectrolyte brush coatings were prepared from these solutions, yielding dry thicknesses of 1.7 ± 0.3 and 2.4 ± 0.1 nm for **P1**‐OSO_3_‐BP and **P2**‐OSO_3_‐BP brushes, respectively (Figure 3a). In contrast, brush formation with **P3**‐BP under optimized conditions produced stable brushes with a dry thickness of approximately 4.3 ± 0.7 nm after extraction. Although higher temperatures during **P3**‐BP self‐assembly are generally entropically unfavourable, they are required to singularize the block copolymer chains, facilitating effective hydrophobic interactions with the substrate. The minor difference in dry thickness before and after extraction supports the directed assembly of the block copolymer chains driven by the hydrophobicity of the BP units. Importantly, ^1^H NMR spectroscopy of **P3** before and after 16 min of UV irradiation under conditions simulating photo‐immobilization confirmed that the photochemical process did not compromise the chemical integrity of the polymer backbone, as suggested by the unchanged integrals of the two samples (Figure S17). These findings align with previous reports indicating that PHEMA does not undergo photodegradation or oxidation upon UV irradiation at 365 nm [46]. The grafting density of both sulfated and non‐sulfated polymers was estimated according to Equation S1 based on the measured dry thickness, with the molecular weights calculated from ^1^H NMR analysis (Table 1), and assuming a polymer density of 1.07 g cm^−3^ for PHEMA [47]. The resulting grafting densities of 0.04 ± 0.01, 0.013 ± 0.001, and 0.08 ± 0.01 chains nm^−2^ for **P1**‐OSO_3_‐BP, **P2**‐OSO_3_‐BP, and **P3**‐BP, respectively (Figure S18a), fall within the typical range reported for grafting‐to approaches [48]. Based on these grafting densities, the average interchain distance (*L*) and the degree of chain overlap, expressed as *L*/2*R*_g_, were calculated according to polymer brush theory and Equation S2 [49]. All the investigated coatings exhibited *L*/2*R*_g_ values below unity, indicating a brush regime with overlapping rather than isolated polymer chains in mushroom conformation (Figure S18b), particularly for polyelectrolyte chains, where electrostatic repulsion further promotes chain extension [50]. Importantly, this demonstrates that all coatings are generally comparable by locating in the brush regime. Nonetheless, within this brush regime, minor variations were observed: **P3**‐BP brushes showed the strongest chain overlap, whereas **P2**‐OSO_3_‐BP brushes exhibited the lowest degree of chain overlap. These variations suggest differences in chain conformational freedom, which may influence the spatial presentation and accessibility of functional groups. For **P1**‐ and **P2**‐OSO_3_‐BP, electrostatic repulsion between charged chains is expected to further promote brush‐like conformations, even at moderate grafting densities. Further surface wettability analysis (Figure 3b) revealed lower water CA values for **P2**‐OSO_3_‐BP (∼30°) than for **P1**‐OSO_3_‐BP (∼45**°**), which was similar to the unsulfated **P3**‐BP control. A decrease in CA upon sulfation is expected [51, 52]. The moderate CA decrease for **P2**‐OSO_3_‐BP and the unaltered CA of **P1**‐OSO_3_‐BP compared to **P3**‐BP might originate from the intrinsic hydrophobicity of the methacrylate backbone and the reduced flexibility of shorter chain polymer brushes. Elevated CA values of 75 ± 3° have been reported for lPGS‐coated PS substrates with a highly flexible, hydrophilic polymer backbone and comparable DS, likely arising from the poor surface coverage obtained in this study [45]. In contrast, CAs below 15° have been reported for brushes containing sulfated sugar units [53]. To exclude UV‐induced desulfation, elemental analysis of UV‐irradiated **P2**‐OSO_3_‐BP—simulating photo‐immobilization conditions—was performed, confirming the sulfate group integrity as the measured values matched the theoretical predictions before and after irradiation (Table S1). To further verify the presence of sulfated polymers on the surface, X‐ray photoelectron spectroscopy (XPS) survey spectra were recorded (Figure S19). A sulfur signal (S 2p) was observed exclusively for the polysulfate‐modified samples, while it was absent in the controls, confirming successful surface modification with the polyelectrolyte brushes. Furthermore, methylene blue staining of Petri dishes coated with **P1**‐ and **P2**‐OSO_3_‐BP, as well as **P3**‐BP, revealed substantial blue staining with the cationic dye exclusively for the sulfated coatings. This confirms the presence and accessibility of the sulfate groups for electrostatic dye binding compared to unsulfated **P3**‐BP‐coated and bare PS controls (Figure 3e).

Overall, despite being thinner than the unsulfated control, our results demonstrate successful preparation of **P1**‐OSO_3_‐BP and **P2**‐OSO_3_‐BP polyelectrolyte brushes via a grafting‐to approach. Notably, the resulting coatings surpass the thicknesses previously reported for grafting‐to assemblies of lPGS‐based block copolymers on PS substrates under comparable salt and polymer concentrations, while eliminating the need for salt pre‐equilibration of the substrates [45].

As the coating's topography, alongside chemical composition, thickness, and wettability, influences the cellular responses, including the adhesion of adherent mammalian cells [54], surface morphologies were investigated using tapping‐mode AFM in air. To assess morphological changes upon sulfation, AFM was performed on **P1**‐OSO_3_‐BP and **P2**‐OSO_3_‐BP and compared to the unsulfated **P3**‐BP control. AFM height and phase images revealed a smooth, homogeneous **P1**‐OSO_3_‐BP brush coating closely resembling that of **P3**‐BP brushes (Figure 3c; Figure S20a–c). Accordingly, the cross‐sectional analysis (Figure 3d) over the whole scanned area showed global surface roughness below the nm range for **P3**‐BP brushes, with a slight increase to the 1–2 nm range for **P1**‐**2**‐OSO_3_‐BP polyelectrolyte brush coatings. However, the surface roughness (< 500 pm) decreased significantly when the analysis was restricted to smaller, 1 µm^2^ areas (Figure S21), confirming that the thickness increase observed in ellipsometry exceeds the local surface roughness. In contrast, **P2**‐OSO_3_‐BP coatings exhibited nanoscale surface aggregates in the topography and phase images (Figures 3c and S20b) with an average diameter of 100–150 nm and a height of up to 20 nm dispersed across the 5 µm^2^ scanned area, according to cross‐section analysis (Figure 3d). However, the detected low contrast in phase images (Figure S20) of both polyelectrolyte brushes reflects low compositional heterogeneity [55], confirming the uniform surface coverage of the coatings. Notably, the few darker areas in **P3**‐BP´s phase images (Figure S20c) may correspond to exposed PS substrate, which was not visible for the sulfated coatings (Figure S20a,b). Larger‐area scans of 20 µm^2^ (Figure S22) revealed additional isolated aggregates (diameter ∼ 0.5 µm, height ∼ 20 nm) in the **P2**‐OSO_3_‐BP brush coatings, which is consistent with the aggregation behavior observed in solution by DLS. Importantly, according to the phase images (Figures S20 and S22), the polyelectrolyte brush coatings appeared to uniformly cover the substrate without patches as observed for lPGS coatings [45]. Besides **P2**‐OSO_3_‐BP, no visible aggregates were detected in **P3**‐BP and **P1**‐OSO_3_‐BP coatings, suggesting that their assembly occurred predominantly via solubilized single chains, as also supported by the DLS data.

To explore the biological performance of the **P1**‐**2**‐OSO_3_‐BP brush coatings with GAG‐mimicking structure, we first assessed cell adhesion and viability of human blood vessel‐related cell types in the presence and absence of serum. GAGs, such as heparan sulfate or heparin, typically mediate cell surface interactions via growth factor binding, stabilization, and local concentration, promoting initial cell adhesion on GAG‐modified substrates. Downstream signaling through receptor binding on the cell surface further promotes cell proliferation and migration [56]. By reducing the structural complexity associated with natural GAGs in protein binding, this fully synthetic approach enables systematic investigation of how chain length and the presence of charges modulate the interactions between GAG‐inspired polymers and serum proteins, including growth factors. Furthermore, cell surface interactions can be probed in vitro in the presence and absence of serum proteins or with defined growth factor supplements. Therefore, the sulfated brush coatings were prepared on PS Petri dishes according to the established protocol, with non‐sulfated **P3**‐BP brushes and pristine TCPS dishes serving as controls. Under standard, serum‐containing culture, both **P1**‐OSO_3_‐BP and **P2**‐OSO_3_‐BP brush coatings supported comparable adhesion and proliferation of human umbilical vein endothelial cells (HUVECs) and human aortic smooth muscle cells (SMCs) to those observed on TCPS controls (Figure S23a,b). Thus, the obvious initial cellular response appears to be independent of the brushes' molecular weight and surface topography. Additional fluorescence imaging of live/dead‐stained HUVECs after 24 h and human dermal fibroblasts (HDFs) after 48 h revealed no evidence of acute cytotoxicity of the polyelectrolyte coatings prepared from **P1**‐OSO_3_‐BP and **P2**‐OSO_3_‐BP (Figure S23a,c). It is well‐documented in literature that charge‐neutral PHEMA‐based coatings can exhibit either antifouling or adhesive behavior depending on chain length, grafting density, and the specific cell type [28]. 24 h after cell seeding, non‐sulfated **P3**‐BP coatings exhibited substantially reduced numbers of adherent cells compared to TCPS controls. Interestingly, the adhesion of HUVECs was even more reduced compared to SMCs and HDFs (Figure S23), resulting in HUVEC‐based spheroid formation and isolated cell patches for SMCs and HDFs on **P3**‐BP brushes. However, by extending the culture time of SMCs and HDFs to 48 h, hardly any cell proliferation was observed, confirming the non‐permissive properties of the prepared **P3**‐BP brushes. Overall, these results confirm the general cell compatibility of the established PHEMA‐based polyelectrolyte coatings with blood vessel‐relevant cell types, promoting cell adhesion and proliferation.

**FIGURE 4:**
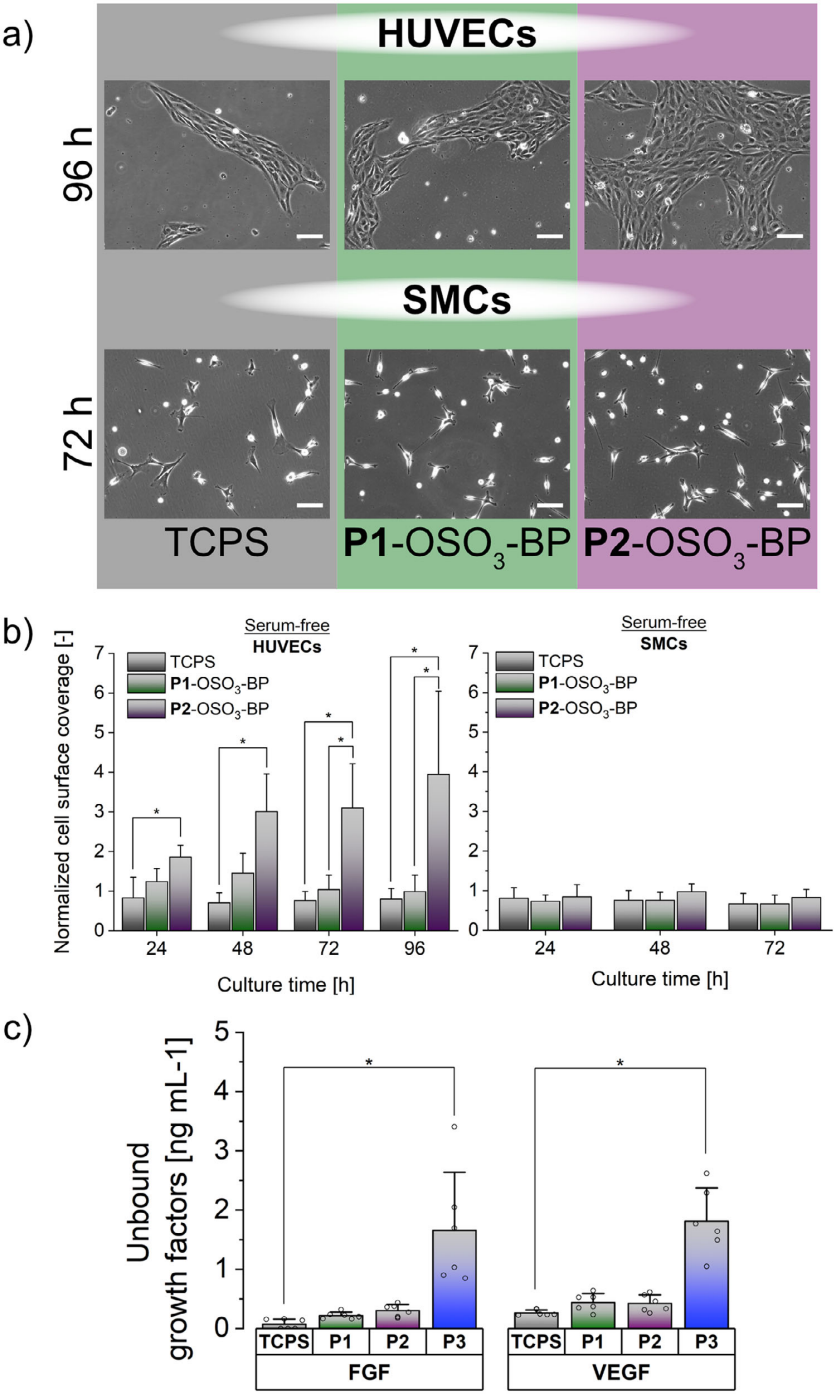
Polyelectrolyte brush coatings (**P1**‐OSO_3_‐BP, **P2**‐OSO_3_‐BP) and TCPS controls in serum‐free medium. (a) Representative phase contrast images of HUVECs and SMCs on day 4 and 2, respectively, after seeding under serum‐free conditions. (b) Time‐dependent cell surface coverage from 24 to 96 h for HUVECs and 24 to 72 h for SMCs, normalized to the respective surface coverage at 4 h under serum‐free conditions. Seeding density: 1 × 10^4^ cells cm^−2^. Scale bar = 100 µm. (c) Residual VEGF and bFGF were detected in the supernatant of **P1**‐OSO_3_‐BP‐ and **P2**‐OSO_3_‐BP‐coated dishes, as well as non‐sulfated **P3**‐BP‐coated dishes and TCPS controls, by ELISA after 2 h incubation with serum‐free, basal cell culture medium supplemented with either VEGF or bFGF (5 ng mL^−1^). (n = 3) Statistical significance was tested with a non‐parametric Kruskal‐Wallis ANOVA test (^*^, *p* < 0.05).

To further investigate electrostatic interactions between anchorage‐dependent mammalian cells and the polymer coating in the context of growth factor binding and delineate potential adhesion mediators, we next evaluated HUVECs’ and SMCs’ adhesion on **P1**‐ and **P2**‐OSO_3_‐BP brush coatings in serum‐free culture using defined growth factor‐supplemented medium. This approach eliminates competitive adsorption of serum proteins onto the charged brushes, thereby preventing RGD‐ and protein‐mediated cell adhesion [57]. The cells were microscopically observed at defined time points after seeding on the sulfated brushes, and compared to pristine TCPS as controls (Figure S24). While no substantial differences could be observed on all substrates after 4 h, the number of adherent HUVECs substantially increased on **P2**‐OSO_3_‐BP brushes within 96 h compared to **P1**‐OSO_3_‐BP and the TCPS control with characteristic healthy cell morphology (Figure 4a). Quantitative analysis of time‐dependent surface coverage by HUVECs on polyelectrolyte brushes and TCPS controls, using automated image analysis of five images per time point across three independent experiments, confirmed this trend (Figure 4b). The combined results suggest a molecular weight‐dependent effect of the polyelectrolyte brushes on HUVEC adhesion and proliferation under serum‐free culture, which was masked in the presence of serum.

In contrast to HUVECs, SMCs showed reduced proliferation on both **P1**‐OSO_3_‐BP and **P2**‐OSO_3_‐BP brush coatings under serum‐free conditions (Figure S24b). Similar observations of reduced SMC adhesion and proliferation were made on heparin and heparin‐like surfaces cultured under ultralow (0.5%) up to high (10%) serum conditions [58, 59, 60]. Furthermore, SMCs on the polyelectrolyte brushes adopted an elongated morphology—characteristic of a contractile phenotype—as expected in the absence of serum [59, 61], exhibiting minimal proliferation within 72 h, similar to their behavior on TCPS (Figure 4a). Quantitative time‐dependent analysis over 72 h confirmed a stable, non‐increasing SMC surface coverage (Figure 4b), a feature beneficial in maintaining vascular tone. The enhanced HUVEC adhesion and proliferation, together with suppressed SMC overgrowth, support the feasibility of sulfated PHEMA as a coating for the selective growth of vascular cell populations. The divergent responses of endothelial versus smooth muscle cells in serum‐free culture suggest that **P1‐2**‐OSO_3_‐BP coatings provide bioactive cues favorable to HUVEC but not SMC adhesion and proliferation. Furthermore, HUVEC proliferation appears to be modulated by the molecular weight and/or corresponding layer thickness of the polyelectrolyte brushes, being more pronounced on the longer‐chain **P2**‐OSO_3_‐BP (65 kDa backbone; 2.4 nm) than on the shorter **P1**‐OSO_3_‐BP (15 kDa; 1.9 nm). We reasoned that this selectivity stems from the GAG‐mimicking nature of the coatings, which may electrostatically sequester and present growth factors − such as VEGF and/or bFGF. While the supplement cocktail for the serum‐free endothelial cell medium includes both growth factors, the corresponding SMC medium supplement contains only bFGF, along with other factors not directly involved in heparin‐mediated signaling.

To verify this hypothesis, we performed an indirect ELISA to quantify residual growth factors in the supernatant after incubating **P1‐2**‐OSO_3_‐BP brush coatings with 5 ng mL^−1^ VEGF‐ or bFGF‐supplemented basal medium for 2 h at 37°C, using non‐sulfated **P3**‐BP brush coatings and pristine TCPS dishes as controls (Figure 4c). After 2 h incubation at 37°C, substantially reduced concentrations in the range of 0.2 – 0.4 ng mL^−1^ were detected for bFGF and VEGF in the supernatants of **P1‐2**‐OSO_3_‐BP‐coated dishes, comparable to those on TCPS, which indicates effective growth factor binding of these substrates. In contrast, the amount of growth factors detected in the supernatants of **P3**‐BP‐coated dishes was considerably higher (1.5 ng mL^−1^), as expected for bioinert coatings and significantly different from TCPS (< 0.2 ng mL^−1^). Interestingly, despite comparable amounts of surface‐bound VEGF and bFGF on polyelectrolyte brush‐coated dishes and TCPS controls according to the ELISA results, only **P2**‐OSO_3_‐BP brushes promoted significant proliferation of HUVECs under serum‐free conditions. This points towards **P2**‐OSO_3_‐BP brush‐induced stabilization of bound growth factors in an active conformation, enhancing their ability to interact with cell surface receptors [62]. Heparin and other sulfated polymers are known to bind and stabilize growth factors such as bFGF and VEGF through specific electrostatic interactions that involve subtle conformational adjustments, thereby facilitating receptor complex formation, dimerization, and downstream signaling activation [63, 64]. Growth factors nonspecifically adsorbed on TCPS seem to denature, losing their bioactivity and thus failing to stimulate cell proliferation, even though their surface‐bound concentrations are comparable.

In contrast to the molecular weight–independent bFGF proliferative response mediated by heparin‐mimicking polyelectrolytes (6.6–80 kDa) in solution [65], VEGF bioactivity was recently reported to be highly sensitive to heparin chain flexibility [66]. Additionally, it is well documented that growth factors can have distinct effects depending on whether they are presented in solution or surface‐bound [62, 67]. In this context, the mode of surface immobilization is critical for preserving growth factor activity. VEGF surface densities as low as 0.1 ng cm^−^^2^ have been reported to be sufficient for VEGF receptor phosphorylation to occur [62]. Surface densities of site‐specifically immobilized single‐chain VEGF homodimers at 0.86 ng cm^−2^ (see supporting information for the calculation) were reported to promote endothelial cell proliferation with maximum activities at 17.2 ng cm^−2^ [67]. Similarly, for bFGF, surface densities in the low ng cm^−2^ range have been reported to effectively promote HUVEC proliferation to confluence within 3 days at a seeding density of 1 × 10^4^ cells cm^−2^ [68]. The quantified growth factor concentrations in the supernatants of our substrates (Figure 4c) correspond to surface densities of 0.5 ± 0.1 ng cm^−2^ for both polyelectrolyte‐coated dishes and TCPS controls. Similar binding of both growth factors to the polyelectrolyte surface is expected, given their comparable affinity for heparin [69, 70]. The detected surface density of VEGF and bFGF is located at the lower end of the concentration range reported in literature to promote endothelial cell proliferation [67, 68]. Consequently, the hypothesis that longer and more flexible polyelectrolyte chains are required for the bioactivity of electrostatically bound VEGF [66] emerges as the most plausible explanation for the observed stronger proliferative effect of **P2**‐OSO_3_‐BP over **P1**‐OSO_3_‐BP coatings. Moreover, the differential response of SMCs and HUVECs on the polyelectrolyte surfaces is attributed to the poor adhesion and the non‐proliferative state of SMCs in serum‐free culture, which makes them less responsive towards bFGF [71].

**FIGURE 5:**
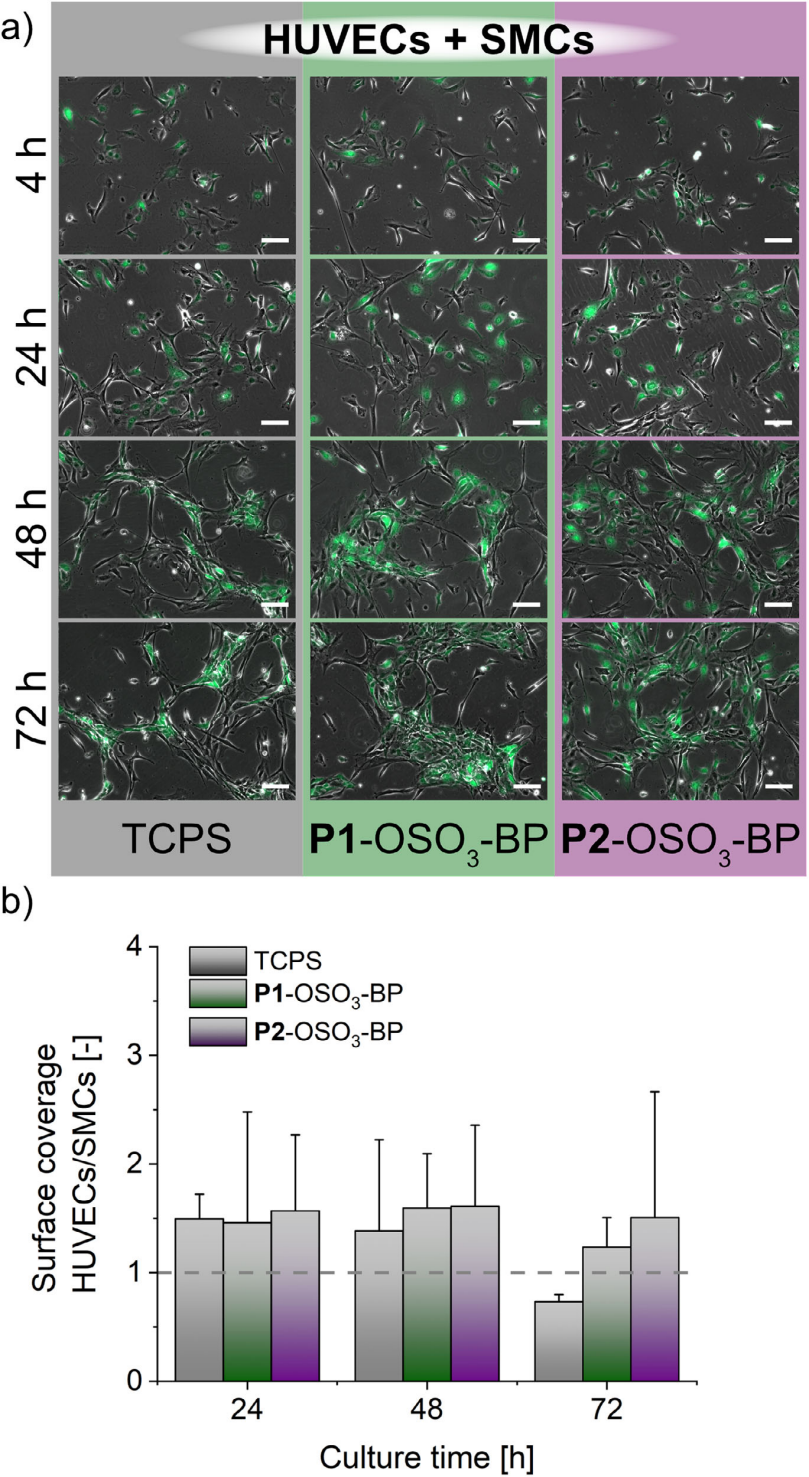
Co‐culture of HUVECs and SMCs on polyelectrolyte brushes (**P1**‐OSO_3_‐BP, **P2**‐OSO_3_‐BP) and TCPS controls in serum‐containing medium. (a) Representative phase contrast and fluorescence images of GFP‐expressing HUVECs (green) and SMCs cultured for 72 h in serum‐containing media (5% FBS). (b) Time‐dependent cell surface coverage ratio of HUVECs/SMCs cultured under the conditions of a), as calculated from automated image analysis. Values at 24, 48, and 72 h were normalized to the 4 h timepoint. The dotted grey horizontal line denotes a HUVEC/SMC surface coverage ratio of 1 (initial 1:1 seeding density). (n = 3).

Based on the selective growth factor binding observed under serum‐free conditions, we next investigated whether these surfaces could also prevent SMC overgrowth in a clinically relevant co‐culture model under serum conditions favoring SMC proliferation (5% FBS). Given that neointimal hyperplasia after implantation of blood‐contacting materials arises from delayed reendothelialization in combination with excessive SMC proliferation, the competitive adhesion and proliferation of HUVECs in direct contact with SMCs were examined. Co‐culture of green fluorescent protein (GFP)‐expressing HUVECs and non‐labeled SMCs on the polyelectrolyte brush‐coated dishes resulted in comparable surface coverage of both cell types over time, with HUVECs/SMCs ratios remaining close to unity (Figure 5). No statistically significant differences were detected between the substrates (Kruskal‐Wallis with Dunn´s post‐hoc test, n = 3 biological replicates) (Figure 5b), indicating that none of the surfaces promoted preferential overgrowth of either cell type. However, a temporal trend was observed. **P2**‐OSO_3_‐BP showed the highest HUVEC/SMC ratio at 48 h, exceeding both TCPS and **P1**‐OSO_3_‐BP. By 72 h, TCPS showed a reduction in the HUVECs/SMCs ratio, suggesting a mild suppression of HUVECs compared to SMC growth, whereas both **P1**‐ and **P2**‐OSO_3_‐BP maintained ratios greater than unity. This indicates that the polymer coatings better preserve HUVEC proliferation under competitive conditions over time. In contrast to the SMC monocultures, which reached confluency within 48 h in the presence of 5% serum (Figure S23b), SMCs in the co‐culture did not reach confluency within 72 h, demonstrating restrained SMC proliferation under competitive conditions. Such modulation of SMC proliferation in the presence of endothelial cells (ECs) has been reported previously in co‐culture systems and is attributed to EC‐derived signaling [72]. At later timepoints, the convergence of surface coverage ratios further indicates a stabilization of EC and SMC growth, consistent with the establishment of a balanced, vessel‐like cellular interface rather than preferential expansion of either cell type, as further confirmed from the persistent colocalization of HUVECs and SMCs across the substrates (Figure 5a).

Having demonstrated the bioactivity of our GAG‐mimetic surfaces, we performed a first proof‐of‐concept experiment to explore the translational potential of this coating to decellularized extracellular matrices (dECMs). Specifically, we applied the coating method to aortic dECM from rats, prepared as described elsewhere [73], to prospectively promote reendothelialization of the dECM scaffold for vascular tissue engineering applications. By selectively enhancing HUVEC adhesion and proliferation while limiting SMC overgrowth, this approach is expected to facilitate rapid and functional endothelialization of clinically relevant scaffolds. Therefore, dECM slices were fixed to supports and functionalized with **P2**‐OSO_3_‐BP using the same protocol as for the plastic substrates. After UV irradiation and water extraction of the unbound chains, treated and untreated dECM slices were incubated overnight in 1 mM methylene blue dye. The strong colour of the functionalized dECM compared to the control (Figure S25) confirmed the presence of multivalent anionic groups, indicating successful immobilization of **P2**‐OSO_3_‐BP polyelectrolyte brushes on dECM. Notably, while control samples showed only weak staining that got removed by repeated washing for one week, the functionalized samples retained their strong coloration under the same washing conditions. This macroscopic observation indicated the presence of covalently immobilized sulfated polymer chains rather than residual, physically adsorbed material. Overall, these findings underscore the feasibility of extending our simple, versatile coating strategy from synthetic surfaces to biologically derived matrices, highlighting its potential for regenerative medicine and blood‐contacting material applications.

## 3 Conclusion

In this work, we present a versatile strategy to fabricate sulfated polyelectrolyte brush coatings from fully synthetic, sulfated PHEMA lacking a carbohydrate backbone, enabling the investigation of charge‐driven interactions inspired by GAG systems. Beyond its simplicity and reproducibility, the use of bioinert, FDA‐approved PHEMA enabled the evaluation of sulfation‐driven biological effects while reducing contributions from the intrinsic bioactivity of natural polysaccharide backbones of GAGs [74, 75]. A DS above 70% imparted strong anticoagulant activity in solution, extending plasma coagulation times beyond 500 s at 0.1 mg mL^−^^1^. Selective solvation under controlled conditions produced largely singularized block copolymers **P1**‐OSO_3_‐BP and **P2**‐OSO_3_‐BP in saline solutions, enabling uniform self‐assembly on PS substrates. Subsequent photoimmobilization via BP anchor groups yielded nm‐thin, smooth polyelectrolyte brushes whose thickness and roughness depended on molecular weight and adsorption conditions. Analysis of the grafting density and chain overlap further confirmed that all the coatings reside in the brush regime, characterized by overlapping polymer chains across all systems. GAG‐inspired bioactivity was confirmed through distinct cell‐type responses. In serum, cell adhesion was strongly enhanced on **P1**‐OSO_3_‐BP and **P2**‐OSO_3_‐BP compared to the cell‐repellent, non‐sulfated **P3**‐BP counterparts. Under serum‐free conditions, only HUVECs proliferated, showing ∼60% surface coverage after 96 h on **P2**‐OSO_3_‐BP brushes. While both polyelectrolyte coatings sequestered comparable amounts of VEGF and bFGF (∼0.5 ng cm^−^^2^), only the longer **P2**‐OSO_3_‐BP chains effectively promoted endothelial proliferation, likely due to their greater chain flexibility, essential for VEGF bioactivity. In contrast, SMC surface coverage remained constant with a contractile morphological phenotype, correlating with minimal responsiveness to sequestered bFGF. Importantly, co‐culture experiments under serum conditions favoring smooth muscle proliferation revealed restrained SMC expansion, stabilization of endothelial growth at later time points, and persistent colocalization of both cell types. This indicates that the coatings maintained a balanced HUVECs/SMCs interface under competitive conditions, contrary to TCPS, which showed a tendency toward endothelial disfavor over time. These results highlight the ability of our system to support the formation of a balanced, vessel‐like cellular biointerface. Together, these findings establish sulfated PHEMA brush coatings as a tunable synthetic platform for promoting reendothelialization at material interfaces in vitro under defined medium composition. In addition to tissue engineering approaches using brush‐functionalized biological scaffolds, future work will focus on establishing and characterizing polymer brushes with varying degrees of sulfation to systematically test surface hemocompatibility, alongside mechanistic studies of receptor activation to clarify how brush molecular weight and chain flexibility regulate the bioactivity of surface‐bound growth factors.

## 4 Experimental Part

The electronic Supporting Information provides a detailed list of materials and methods, and polymer characterization.

## Consent

All authors agree to the final version of this manuscript submitted for publication.

## Conflicts of Interest

The authors declare no conflicts of interest.

## Supporting information




**Supporting File**: mabi70201‐sup‐0001‐SuppMat.docx.

## Data Availability

The data that support the findings of this study are openly available via Zenodo, https://doi.org/10.5281/zenodo.19597115.
