# Supplementary material for: Fully Synthetic, Biomimicking Polysulfates With Tunable Anticoagulant and Endothelial Cell–Selective Bioactivity
Source: Macromol Biosci. 2026 Jun 7;26(6):e70201. doi: 10.1002/mabi.70201 (PMC13242835; doi:10.1002/mabi.70201)
Supplement: Supplementary file 1 — Supporting File: mabi70201‐sup‐0001‐SuppMat.docx. [file MABI-26-e70201-s001.docx]

Supporting Information

Fully Synthetic, Biomimicking Polysulfates with Tunable Anticoagulant and Endothelial Cell–Selective Bioactivity

Andrea Cosimi,^1,2^ Roxana Pollehn,^1^ Andrea De Martino,^1^ Scarlett Manzke,^1^ Yannic Kerkhoff,^1,3^ Philip Nickl,^1^ Theresa Lohmann,^4^ Eriselda Keshi, ^4,5^ Cornelia Lee-Thedieck,^6^ and Marie Weinhart^1,2^*

^1^ Institute of Chemistry and Biochemistry – Organic Chemistry, Freie Universität Berlin,
Takustraße 3, 14195 Berlin, Germany; E-mail: [marie.weinhart@fu-berlin.de](mailto:marie.weinhart@fu-berlin.de)

^2^ Institute of Physical Chemistry and Electrochemistry, Leibniz Universität Hannover, Callinstraße 3A, 30167 Hannover, Germany; E-mail: [marie.weinhart@pci.uni-hannover.de](mailto:marie.weinhart@pci.uni-hannover.de)

^3^ IT & Data Services, Zuse Institute Berlin, Takustraße 7, 14195 Berlin, Germany.

^4^ Department of Surgery, Campus Charité Mitte | Campus Virchow-Klinikum, Experimental Surgery, Charité - Universitätsmedizin Berlin, corporate member of Freie Universität Berlin, Humboldt-Universität zu Berlin, Augustenburger Platz 1, 13353, Berlin, Germany

^5^ Berlin Institute of Health (BIH), Berlin, Germany

^6^ Institute of Cell Biology and Biophysics, Leibniz Universität Hannover,

Herrenhäuser Straße 2, 30419 Hannover, Germany, E-mail: [lee-thedieck@cell.uni-hannover.de](mailto:lee-thedieck@cell.uni-hannover.de)

# Contents

[Contents 2](#_Toc231545554)

[1. Materials 3](#_Toc231545555)

[1.1 Materials for Synthesis 3](#_Toc231545556)

[1.2 Materials for Surface Modification 3](#_Toc231545557)

[1.3 Materials for Cell Culture 4](#_Toc231545558)

[1.4 Materials for Activated Partial Thromboplastin Time (aPTT) 5](#_Toc231545559)

[1.5 Materials for Enzyme-linked Immunosorbent Assay (ELISA) 5](#_Toc231545560)

[2. Synthesis and Characterization 6](#_Toc231545561)

[2.1 Benzophenone Methacrylate (MABP) 6](#_Toc231545562)

[2.1 General Procedure for Sequential RAFT Polymerization 7](#_Toc231545563)

[2.2 Synthesis and Characterization of **P1** 7](#_Toc231545564)

[2.3 Synthesis and Characterization of **P2** 9](#_Toc231545565)

[2.4 Synthesis and Characterization of **P3** 9](#_Toc231545566)

[2.5 Synthesis and Characterization of **P1**-BP 10](#_Toc231545567)

[2.6 Synthesis and Characterization of **P2**-BP 12](#_Toc231545568)

[2.7 Synthesis and Characterization of **P3**-BP 13](#_Toc231545569)

[2.8 General Procedure for Sulfation of **P1-2** and **P1-2**-BP 15](#_Toc231545570)

[2.9 Synthesis and characterization of **P1**-OSO_3_-BP 15](#_Toc231545571)

[2.10 Synthesis and Characterization of **P2**-OSO_3_-BP 17](#_Toc231545572)

[3. Methods for Surface Modification and Characterization 18](#_Toc231545573)

[3.1 Spin Coating of PS Model Substrates 18](#_Toc231545574)

[3.2 Surface Functionalization and Characterization 18](#_Toc231545575)

[3.3 Polymer Self-Assembly via QCM-D 19](#_Toc231545576)

[3.4 Cell Culture 20](#_Toc231545577)

[3.5 Activated Partial Thromboplastin Time (aPTT) 21](#_Toc231545578)

[3.6 Adsorption of VEGF and bFGF 21](#_Toc231545579)

[4. Polymer and Surface Characterization 22](#_Toc231545580)

[4.1 Nuclear Magnetic Resonance (NMR) Spectroscopy 22](#_Toc231545581)

[4.2 Gel Permeation Chromatography (GPC) 22](#_Toc231545582)

[4.3 Combustion Analysis 23](#_Toc231545583)

[4.4 Spectroscopic Ellipsometry (SE) 23](#_Toc231545584)

[4.5 Water Contact Angle (CA) 24](#_Toc231545585)

[4.6 Dynamic Light Scattering (DLS) 24](#_Toc231545586)

[4.7 Optical Microscopy 24](#_Toc231545587)

[4.8 Atomic Force Microscopy (AFM) 25](#_Toc231545588)

[4.9 X-Ray Photoelectron Spectroscopy (XPS) 25](#_Toc231545589)

[5.0 Estimation of scVEGF surface density from reference [3][4] 26](#_Toc231545590)

[5. Supplementary Figures 26](#_Toc231545591)

# Materials

## Materials for Synthesis

Ethanol (EtOH, absolute, ≥ 99%), methanol (≥ 99.8%), toluene (≥ 99.8%), and dichloromethane (DCM) were purchased from Fisher Scientific GmbH (Schwerte, Germany). Technical grade EtOH (Sigma Aldrich, Steinheim, Germany), used for surface preparation, was distilled under reduced pressure to remove impurities. Hydroxyethyl methacrylate (HEMA) (used after cryogenic vacuum distillation to remove hydroquinone monomethyl ether (MEHQ) as the inhibitor), SO_3_∙pyridine (97%), triethylamine (TEA), and 4,4´-azobis (4-cyanovaleric acid) (ACVA) were supplied by Sigma Aldrich (Steinheim, Germany). Sodium hydroxide (NaOH, ≥98%) and pre-wetted regenerated cellulose dialysis tubes (molecular weight cut-off (MWCO): 1 kDa, Spectra/Por® 6) from SpectrumLabs were purchased from Roth (Karlsruhe, Germany). 4‑cyano-4-(phenylcarbonothioylthio)pentanoic acid (CPADB) and 4-hydroxybenzophenone (4-HBP, 98%) were purchased from TCI GmbH (Eschborn, Germany). 1,4-Dioxane (stabilized with butylated hydroxytoluene) was supplied by Grüssing GmbH (Filsum, Germany). Methacryloyl chloride was purchased from ABCR (Karlsruhe, Germany).

## Materials for Surface Modification

Ultrapure water for surface modification and washing was prepared via a Merck Millipore^TM^ water treatment system, Milli-Q, with a minimum resistivity of 18.2 MΩ cm (25 °C). For surface characterization, silicon wafers with a 2 nm SiO_2_ layer supplied by Silchem GmbH (Freiberg, Germany) were cut into square pieces (11 x 11 mm), washed with EtOH, and dried under a stream of N_2_. Gold-coated QCM-D sensor chips (11 mm diameter) were supplied by Q-Sense LOT-Quantum Design GmbH (Darmstadt, Germany). The polystyrene (PS) solution (1 wt-% in toluene) used for spin-coating of the silicon wafers/gold sensors was prepared using commercial PS (*M*_n_= 132 g/mol, *Ð* = 1.9) from Falcon^®^ culture dishes supplied by Th. Geyer GmbH + Co. KG (Berlin, Germany). Aqueous ethanolic solutions of **P3**-BP (0.25 mg mL^-1^) were prepared by first dissolving the solid polymer in EtOH_abs_ overnight and then adding Milli-Q water to reach 10% EtOH (v/v). Solvents used for QCM-D analysis were degassed in an ultrasonic bath for 30 min prior to use. For QCM-D experiments in selective solvents, **P3** and **P3**-BP solutions (0.25 mg mL^-1^) were prepared analogously, while samples in pure EtOH served as controls in a non-selective solvent. **P1**-OSO_3_-BP (0.75 mg mL^-1^, 2.6 x 10^-5^ M) and **P2**-OSO_3_-BP (0.55 mg mL^-1^, 5.3 x 10^-6^ M) solutions in 0.8 M NaCl were prepared by dilution of polymer stock solutions (2 mg mL^-1^) in Milli-Q water with 3 M NaCl stock solutions. For salt-free controls, **P1**-OSO_3_-BP and **P2**-OSO_3_-BP were dissolved in Milli-Q water at concentrations of 0.75 and 0.55 mg mL^-1^, respectively. Methylene blue (Merck, Darmstadt, Germany) solutions used for staining **P1**-OSO_3_-BP- and **P2**-OSO_3_-BP-coated Petri dishes were prepared by dissolving the solid dye powder in Milli-Q water to a final concentration of 1 mM.

## Materials for Cell Culture

Falcon^®^ PS culture dishes (Ø 35 mm) were purchased from Th. Geyer GmbH + C. KG (Berlin, Germany). Tissue culture PS dishes (Ø 35 mm) were supplied by VWR International (Leuven, Belgium). Dulbecco’s modified Eagle medium (DMEM) with 4.5 g L^-1^ glucose, 1% penicillin-streptomycin, trypsin/EDTA solution (0.05%), and Dulbecco’s phosphate-buffered saline solution containing CaCl_2_ and MgCl_2_ (DPBS) was purchased from Thermo Fisher Scientific (Darmstadt, Germany). Endothelial cell growth medium (VascuLife®VEGF - Endothelial Cells Growth Medium Kit; Cat.#: LL-0003), smooth muscle growth medium (VascuLife®SMC - Smooth Muscle Cells Growth Medium Kit; Cat.#: LL-0014), human umbilical vein endothelial cells (HUVECs), and human aortic smooth muscle cells (SMCs) were purchased from CellSystems GmbH (Troisdorf, Germany). Green fluorescent protein (GFP)-tagged HUVECs were purchased from Cellworks (Caltag Medsystems Company). Human dermal fibroblasts (HDFs) were isolated and expanded according to established procedures.[1] According to the manufacturer’s instructions, 2% FBS (included in the VascuLife®VEGF LifeFactors Kit) was added to the VascuLife^®^ VEGF medium together with the other provided supplements (ascorbic acid (50 µg mL^-1^), hydrocortisone hemisuccinate (1 µg mL^-1^), L-glutamine (10 mM), rh IGF-1(15 ng mL^-1^), rh EGF (5 ng mL^-1^), heparin sulfate (0.75 U mL^-1^), rh bFGF (5 ng mL^-1^), and VEGF (5 ng mL^-1^)) for cell culture experiments in serum-containing media. For serum-free experiments, the VascuLife^®^ VEGF medium was prepared accordingly without FBS addition. Similarly, for SMC culture, 5% FBS (included in the VascuLife®SMC LifeFactors Kit) was added to the VascuLife^®^ SMC medium together with the other provided supplements (ascorbic acid (50 µg mL^-1^), L-glutamine (10 mM), rh insulin (5 µg mL^-1^), rh EGF (5 ng mL^-1^), rh bFGF (5 ng mL^-1^)) for cell culture experiments in serum-containing media. For serum-free experiments, the VascuLife^®^ SMC medium was prepared accordingly without FBS addition. For HUVEC/SMC co-culture, a 1:1 mixture of the VascuLife^®^ VEGF medium and VascuLife^®^ SMC medium (both serum-free) was prepared and supplemented with 5% FBS (included in the VascuLife^®^ SMC LifeFactors Kit). For HDF culture, fetal bovine serum (FBS) was purchased from PAN-Biotech GmbH (Aidenbach, Germany). Propidium iodide (PI) and fluorescein diacetate (FDA) were supplied by Sigma Aldrich (Steinheim, Germany).

## Materials for Activated Partial Thromboplastin Time (aPTT)

Lyophilized standard human plasma (#ORKL17), actin FS Dade® Actin® FS Activated PTT Reagent (#10445712), and CaCl_2_ (#ORHO37) were purchased from Siemens Healthineers (Forchheim, Germany). Unfractionated heparin (UFH) (#SLBN2208V, 201 USP mg^-1^ dry basis) was purchased from Sigma Aldrich (Steinheim, Germany).

## Materials for Enzyme-linked Immunosorbent Assay (ELISA)

ELISA kits (# KHG0111 for VEGF; # KHG0021 for bFGF) were purchased from Invitrogen, Thermo Fisher Scientific (Schwerte, Germany). Tissue culture PS (TCPS) dishes (Ø 35 mm) were supplied by VWR International (Leuven, Belgium). Falcon^®^ PS culture dishes (Ø 35 mm) were purchased from Th. Geyer GmbH + C. KG (Berlin, Germany).

# Synthesis and Characterization

## Benzophenone Methacrylate (MABP)

**Scheme S1.** Synthesis of the photo-reactive comonomer MABP.

The photoreactive MABP was synthesized according to *Schweigerdt* et al..[2]

**^1^H-NMR** (400 MHz; CDCl_3_) δ = 7.81-7.17 (m, 10H), 6.31 (m, 1H), 5.74 (m, 1H), 2.01 (m, 3H) ppm.


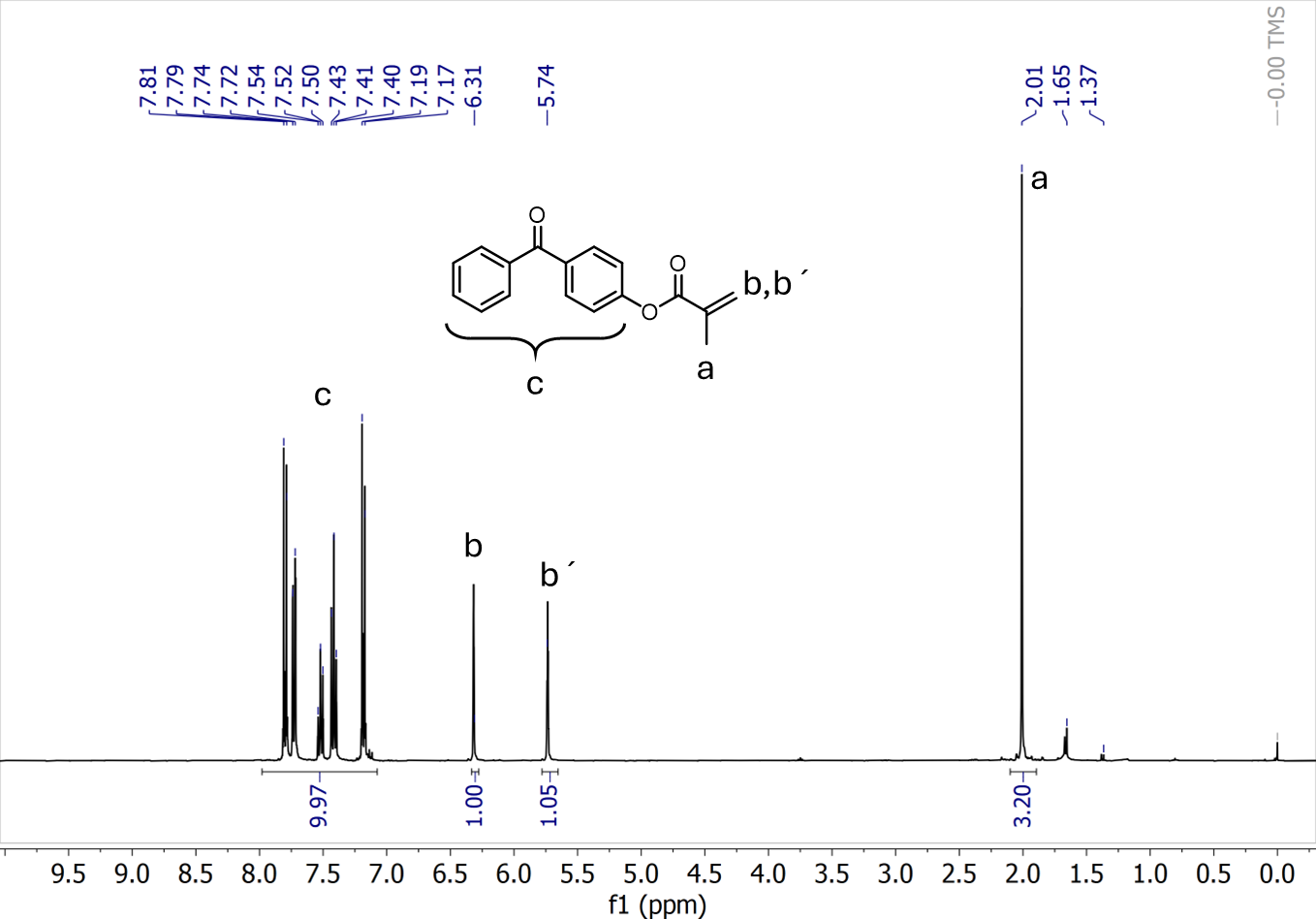


**Figure S1.** ^1^H-NMR of the photo-reactive monomer MABP recorded at 400 MHz in CDCl_3_.

## General Procedure for Sequential RAFT Polymerization

After cryogenic vacuum distillation, HEMA was placed in a one-neck round-bottom flask together with ACVA (0.2 eq with respect to the HEMA monomer) and CPADB (1 eq) and was dissolved in a mixture of water:1.4-dioxane. The resulting solution was degassed for 30 minutes by Ar flushing. The reaction was initiated by placing the flask in a pre-heated oil bath at 70 °C and stirred under these conditions for 16 h under inert atmosphere. The reaction was quenched by cooling the flask with an ice bath. The resulting PHEMA was concentrated under reduced pressure, dissolved in MeOH, and dialyzed against MeOH with daily solvent exchange for 3 d. After solvent removal, the pure PHEMA homopolymers **P1**, **P2**, and **P3** were obtained as pink crystalline solids.

For sequential RAFT, the crude polymers were converted *in situ* with BP-based comonomers to **P1**-, **P2**-, and **P3**-BP block copolymers. For that, ACVA (0.2 eq) and BP were dissolved in 1,4-dioxane. This solution was added to the ice-cold PHEMA mixture. After degassing for 30 minutes by Ar flushing, the reaction was reinitiated at 70 °C and stirred for 16 h in the absence of light. The reaction was quenched by cooling down in an ice bath and exposure to air. The raw mixture was concentrated, dissolved in MeOH, and dialyzed against MeOH (MWCO 1 kDa) with daily solvent exchange for 3 d. After solvent removal, the pure copolymers were obtained as pink solids.

Synthesis of polymers **P1**, **P2**, and **P3** was carried out as described above, with HEMA eq adjusted according to the targeted molecular weight. BP eq were adjusted accordingly for the polymers **P1**-, **P2**-, and **P3**-BP´s targeted repeating units (r.u.).

## Synthesis and Characterization of **P1**

HEMA = 5.5 g, (42.2 mmol, 75 eq.), ACVA = 31.6 mg (0.1 mmol, 0.2 eq.), CPADB (157.4 mg, 0.56 mmol, 1 eq.), water:1,4-dioxane (1:1) = 18 mL.


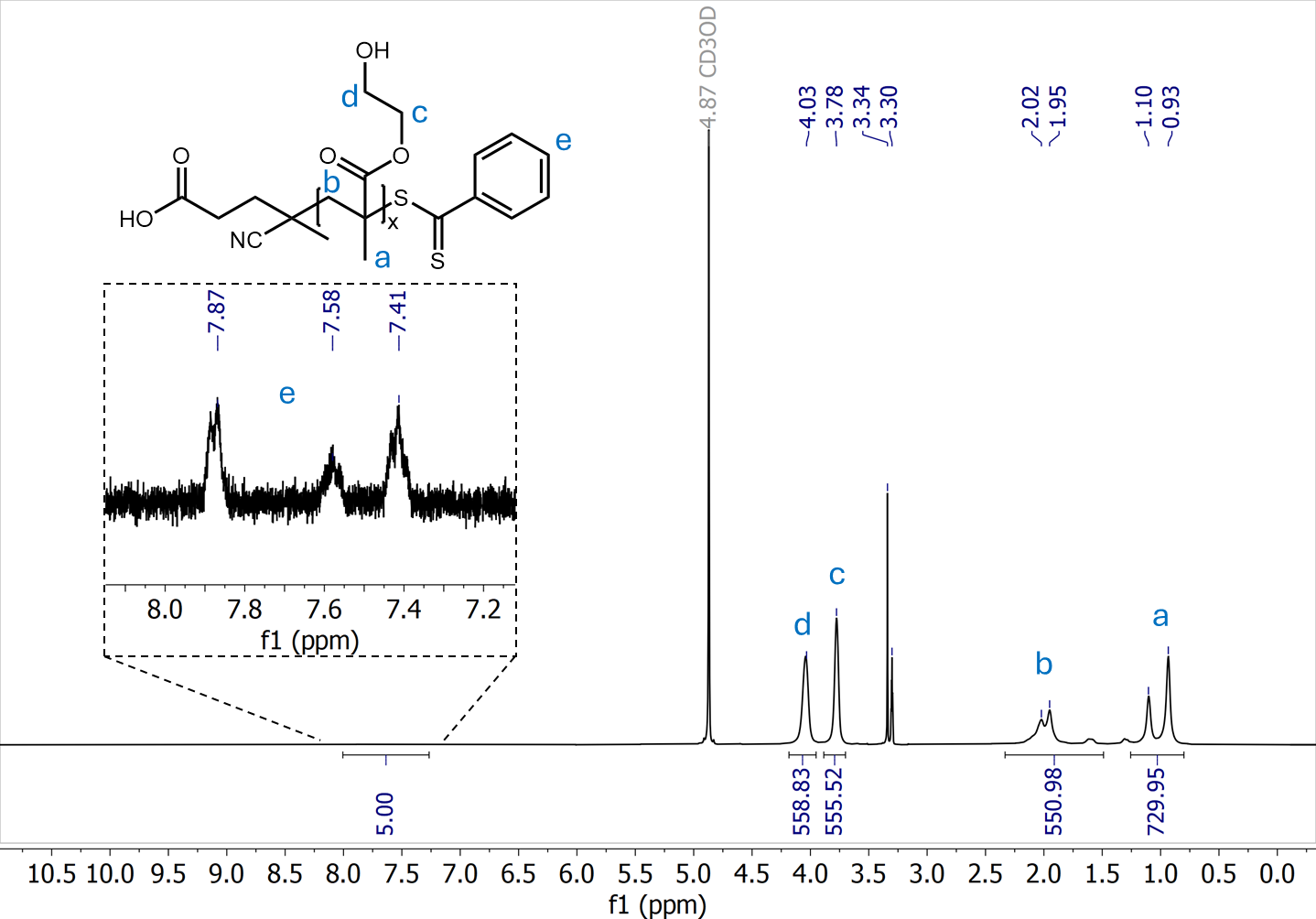


**Figure S2.** ^1^H-NMR spectrum of the homopolymer **P1** recorded at 400 MHz in CD_3_OD.

**^1^H NMR** (400 MHz; CD_3_OD): δ (ppm) = 7.89 – 7.44 (m, 5H), 4.07 (m, 238H), 3.80 (m, 238H), 3.37 – 3.33 (MeOD), 2.05 – 1.98 (m, 242H), 1.13 – 0.96 (m, 360H).

**GPC** (10 mM LiBr in DMF, PMMA standard) = 30760 Da, *Ð* = 1.41

The content of HEMA r.u. in the **P1** polymer was calculated from the ^1^H-NMR spectrum by first referencing the peaks’ integral of the aromatic protons between 7.89 – 7.44 ppm to 5 protons (corresponding to the aromatic protons characteristic of CPADB). The ratio of the integral value *I*_7.89-7.44_ of the aromatic signals and the integral *I*_4.07_ corresponding to 2 methylene protons next to the hydroxyl group per incorporated HEMA monomer yields the number of r.u.’s according to the following equation.

HEMA r.u. = [(*I*_7.89-7.44_/5) x (*I*_4.07_/2)] = 119

## Synthesis and Characterization of **P2**

HEMA = 5.5 g, (42.2 mmol, 385 eq.), ACVA = 6.2 mg (22 µmol, 0.2 eq.), CPADB (30.7 mg, 0.1 mmol, 1 eq.), water:1,4-dioxane (1:1) = 18 mL.


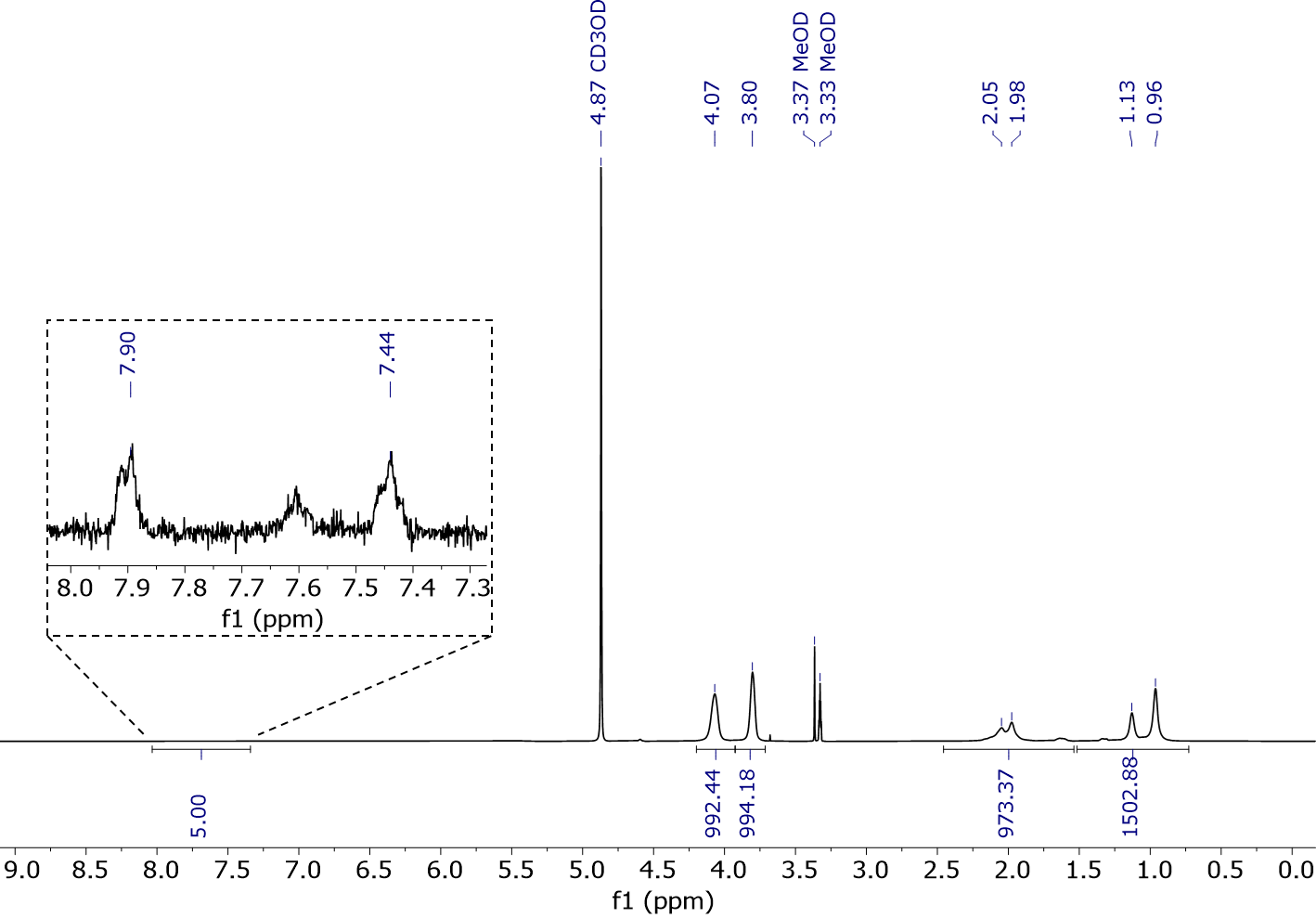


**Figure S3.** ^1^H-NMR spectrum of the homopolymer **P2** recorded at 400 MHz in CD_3_OD.

**^1^H NMR** (400 MHz; CD_3_OD): δ (ppm) = 7.90 – 7.44 (m, 5H), 4.07 (m, 992H), 3.80 (m, 994H), 3.37 – 3.33 (MeOD), 2.05 – 1.98 (m, 973H), 1.13 – 0.96 (m, 1502H).

**GPC** (10 mM LiBr in DMF, PMMA standard) = 128210 Da, *Ð* = 1.31

HEMA r.u. = [(*I*_7.90-7.44_/5) x (*I*_4.07_/2)] = 496

- 1. *Synthesis and Characterization of* ***P3***

HEMA = 2.0 g, (15.4 mmol, 228 eq.), ACVA = 3.8 mg (14 µmol, 0.2 eq.), CPADB (18.8 mg, 67 µmol, 1 eq.), water:1,4-dioxane (1:1) = 7.8 mL.


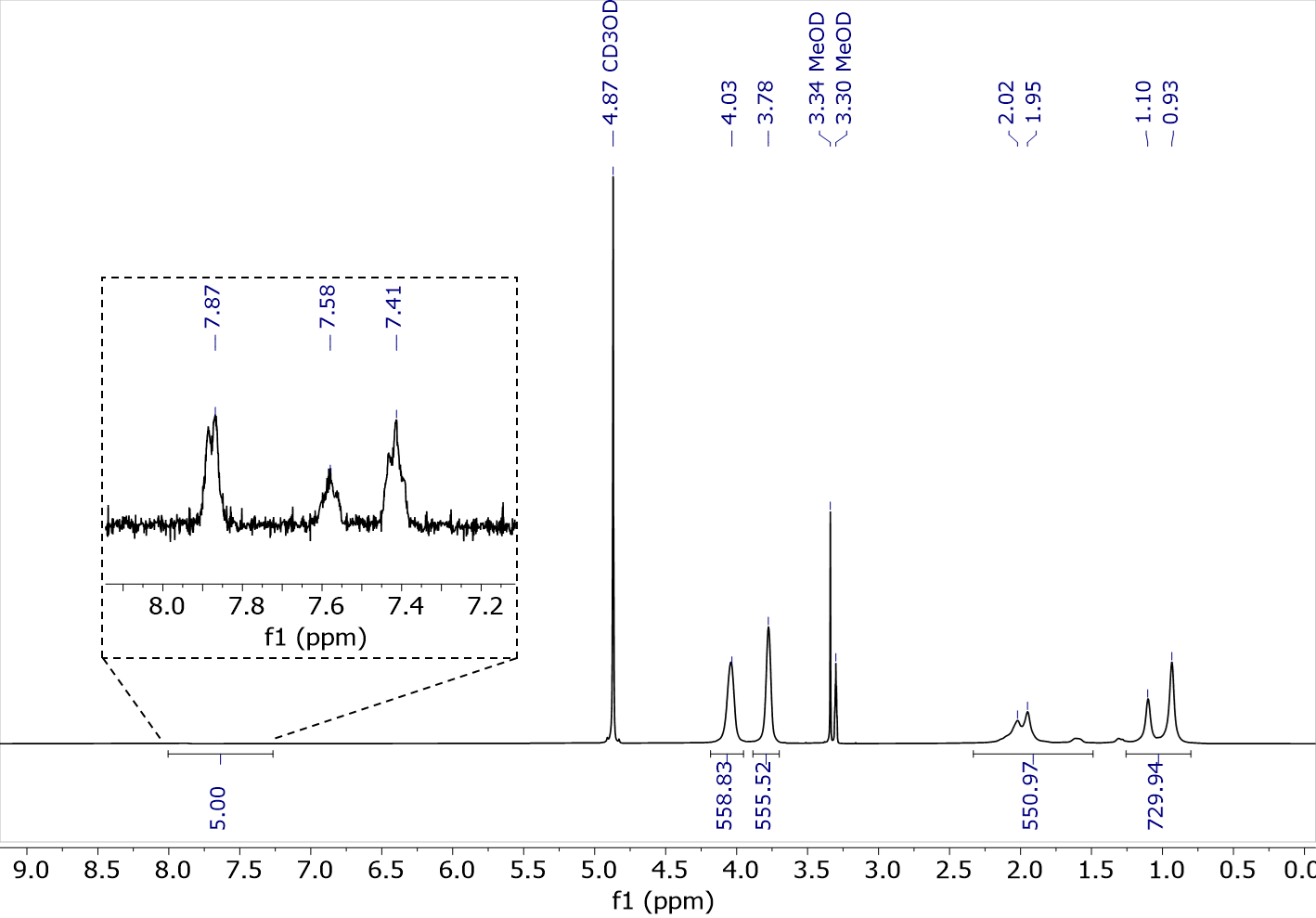


**Figure S4.** ^1^H-NMR spectrum of the homopolymer **P3** recorded at 400 MHz in CD_3_OD.

**^1^H NMR** (400 MHz; CD_3_OD): δ (ppm) = 7.87 – 7.41 (m, 5H), 4.03 (m, 559H), 3.78 (m, 555H), 3.34 – 3.30 (MeOH), 2.02 – 1.95 (m, 550H), 1.10 – 0.93 (m, 730H).

**GPC** (10 mM LiBr in DMF, PMMA standard) = 54694 Da, *Ð* = 1.22

HEMA r.u. = [(*I*_7.87-7.41_/5) x (*I*_4.03_/2)] = 280

- 1. *Synthesis and Characterization of* ***P1****-BP*

**P1** = 5 g (38.4 mmol, 75 eq.), ACVA = 28.7 mg (0.1 mmol, 0.2 eq.), BPMA (545.2 mg, 2.0 mmol, 7 eq.), water:1,4-dioxane (1:1) = 6 mL, yield = 65%

**
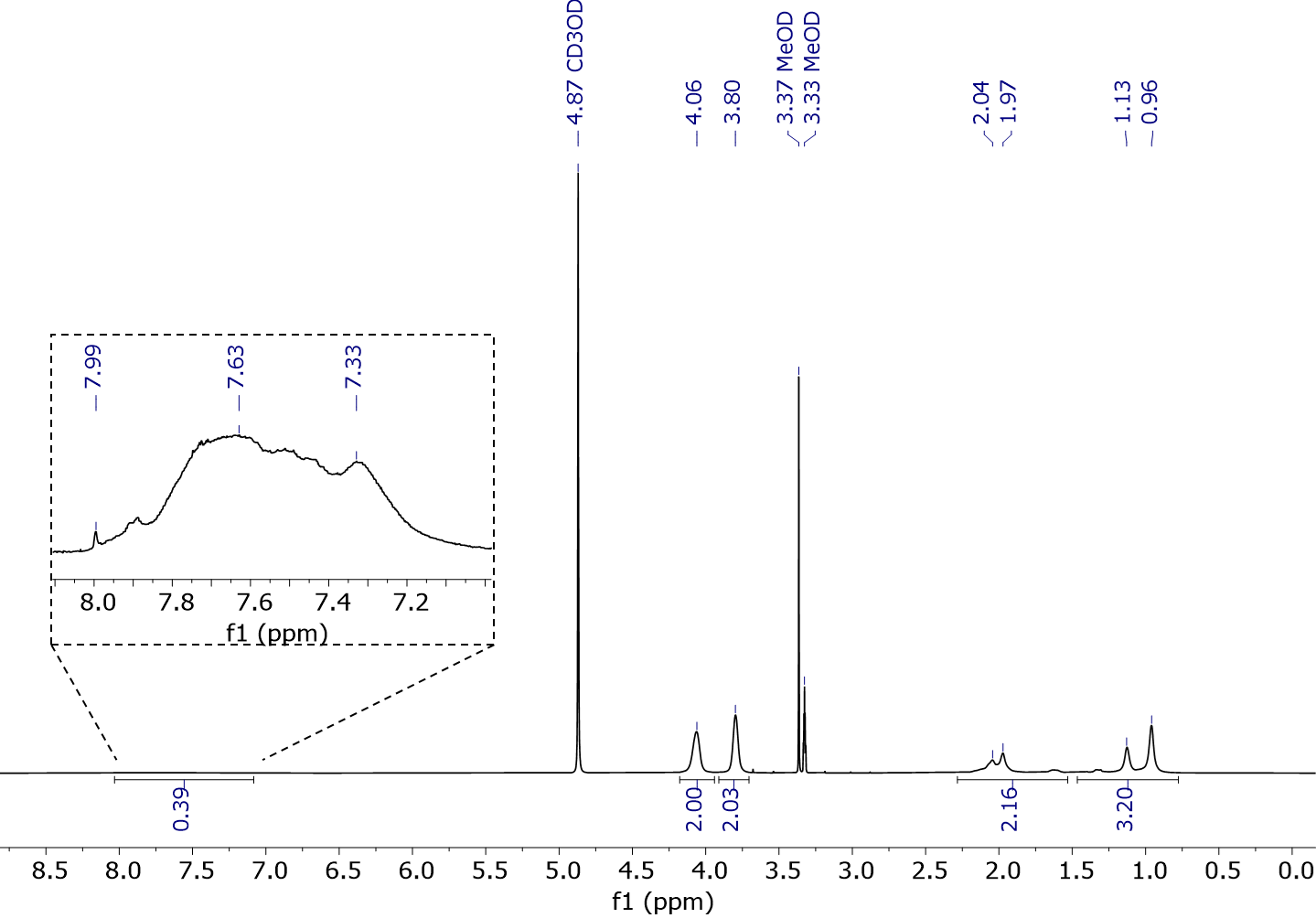
**

**Figure S5.** ^1^H-NMR spectrum of the homopolymer **P1**-BP recorded at 400 MHz in CD_3_OD.

**^1^H NMR** (400 MHz; CD_3_OD): δ (ppm) = 7.99 – 7.33 (m, 0.4H), 4.06 (m, 2H), 3.80 (m, 2H), 3.37 – 3.33 (MeOD), 2.04 – 1.97 (m, 2H), 1.13 – 0.96 (m, 3H).

**GPC** (10 mM LiBr in DMF, PMMA standard) = 35017 Da, *Ð* = 1.29


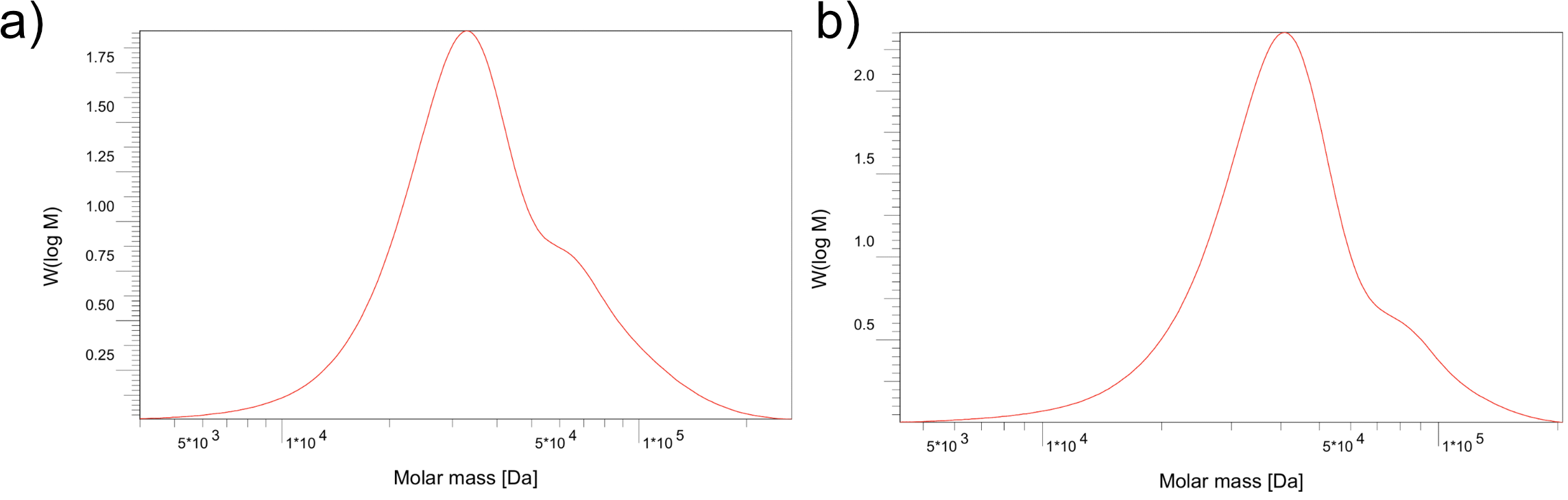


**Figure S6.** GPC chromatograms of **a) P1** homopolymer and **b) P1**-BP block copolymer recorded in DMF (PMMA calibration).

The number of BP r.u.s in the **P1**-BP polymer was calculated as follows: in the ^1^H NMR spectrum of **P1** and **P1**-BP, the peaks’ integral of the protons at 4.07 ppm was referenced to 2 protons (corresponding to the protons characteristic of HEMA r.u.). The integral value of the aromatic region of **P1** (representing the CPADB fragment) was subtracted from the value of the integral in the aromatic signals in **P1**-BP. The difference was then divided by the number of aromatic protons in the BP comonomer (9H), yielding the corresponding average BP units per HEMA r.u.. This value was then multiplied by the number of HEMA r.u.s of **P1** (119) to yield, on average, ~ 5 BP units per block copolymer **P1**-BP.

- 1. *Synthesis and Characterization of* ***P2****-BP*

**P2** = 5 g (38.4 mmol, 75 eq.), ACVA = 5.6 mg (0.02 mmol, 0.2 eq.), BPMA (186.4 mg, 0.7 mmol, 8 eq.), water:1,4-dioxane (1:1) = 6 mL, yield = 68%


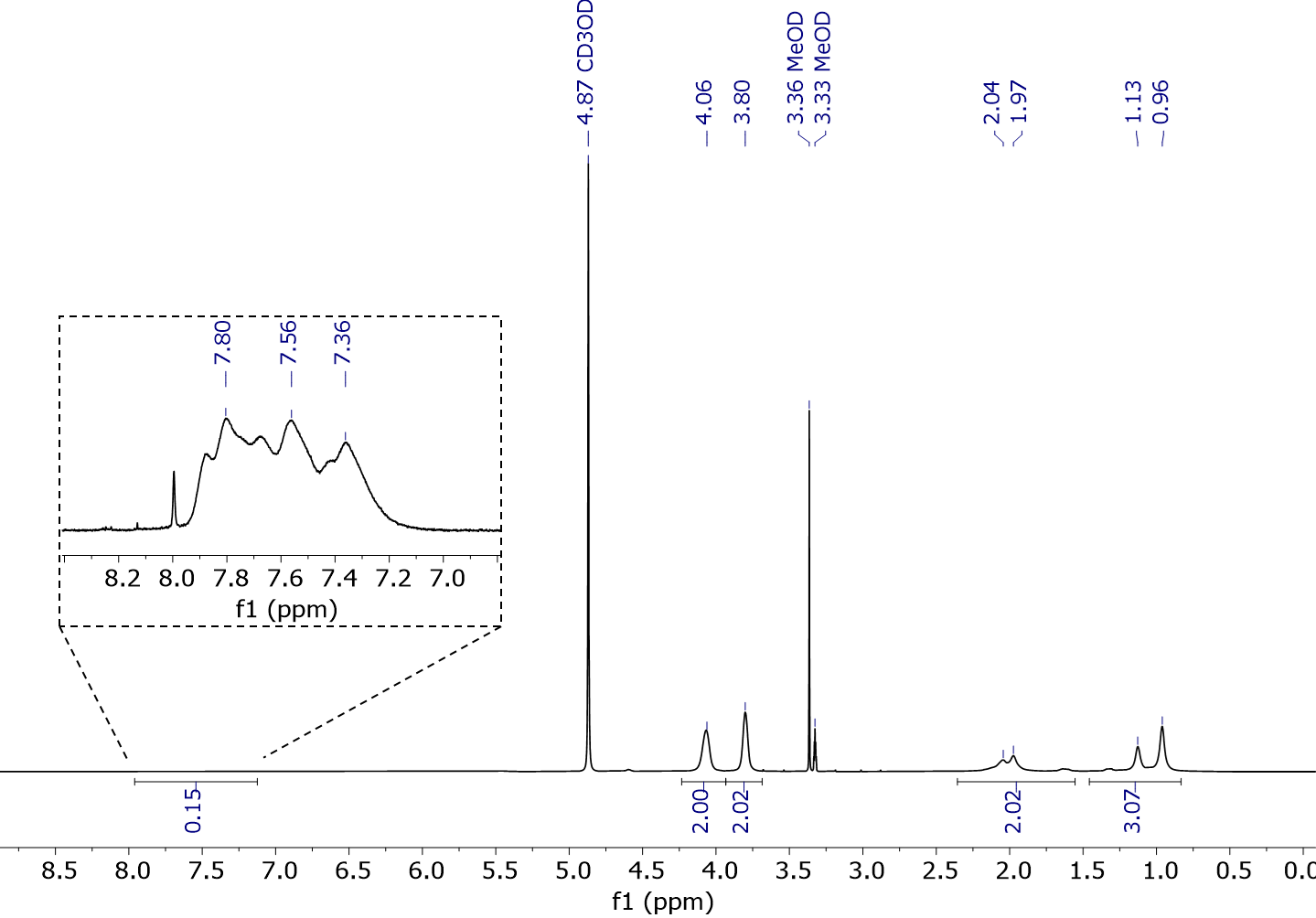


**Figure S7.** ^1^H-NMR spectrum of the homopolymer **P2**-BP recorded at 400 MHz in CD_3_OD.

**^1^H NMR** (400 MHz; CD_3_OD): δ (ppm) = 7.80 – 7.36 (m, 0.15H), 4.06 (m, 2H), 3.80 (m, 2H), 3.36 – 3.33 (MeOD), 2.04 – 1.97 (m, 2H), 1.13 – 0.96 (m, 3H).

**GPC** (10 mM LiBr in DMF, PMMA standard) = 141910 Da, *Ð* = 1.39


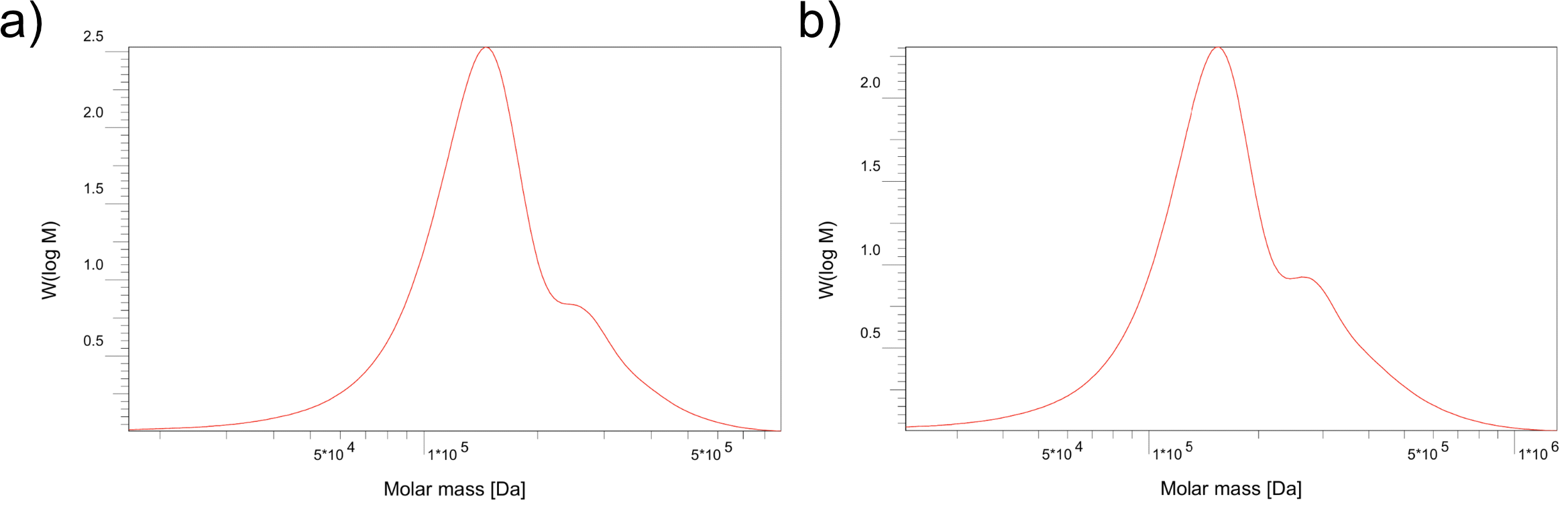


**Figure S8.** GPC chromatograms of **a) P2** homopolymer and **b) P2**-BP block copolymer recorded in DMF (PMMA calibration).

- 1. *Synthesis and Characterization of* ***P3****-BP*

**P3** = 1.7 g (13.3 mmol, 228 eq.), ACVA = 2.8 mg (10 µmol, 0.2 eq.), BPMA (126.1 mg, 0.47 mmol, 7 eq.), water:1,4-dioxane (1:1) = 3 mL, yield = 75%


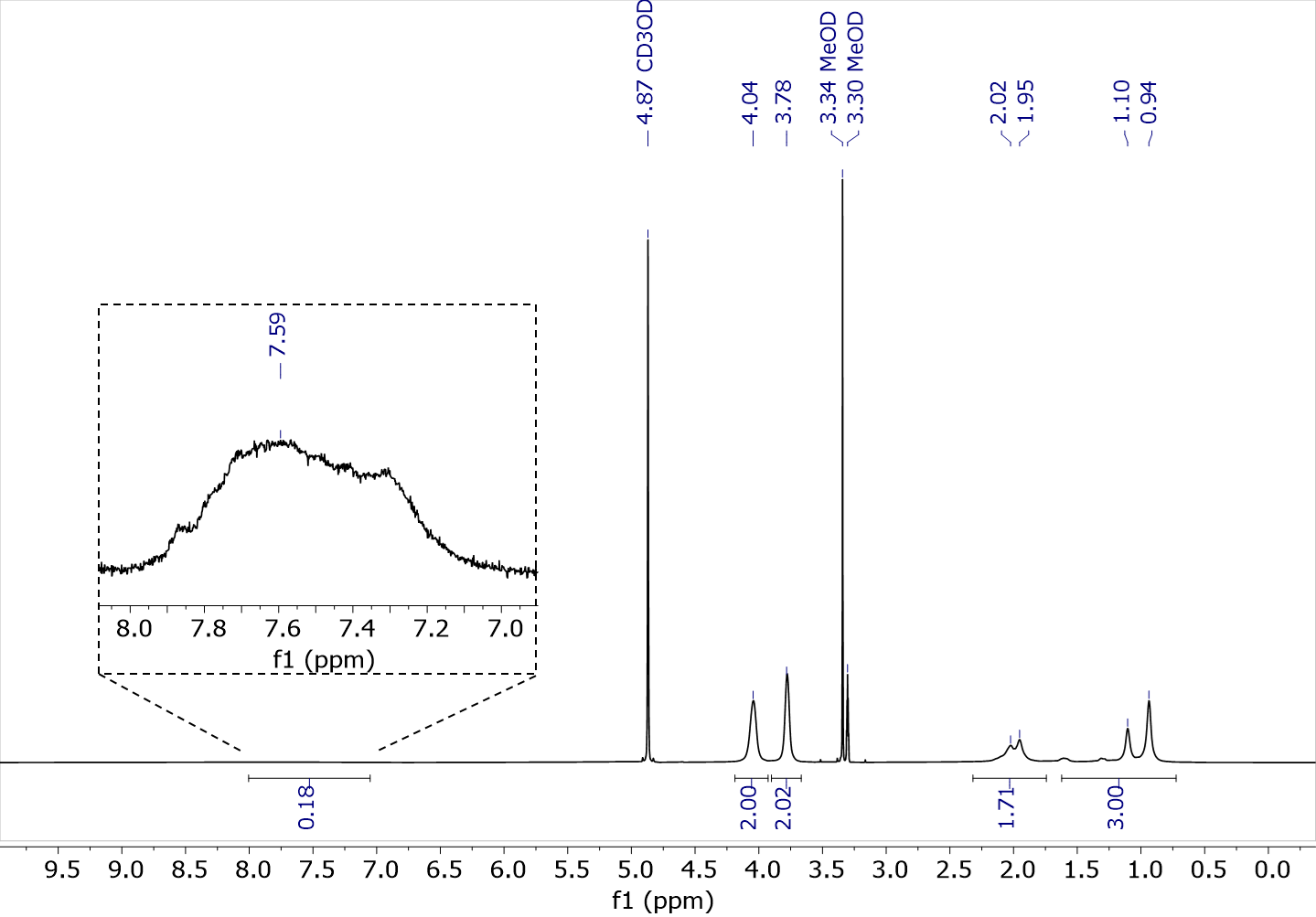


**Figure S9.** ^1^H-NMR spectrum of the photo-reactive block copolymer **P3**-BP recorded at 400 MHz in CD_3_OD.

**^1^H NMR** (400 MHz; CD_3_OD): δ (ppm) = 7.59 (m, 0.18H), 4.04 (m, 2H), 3.78 (m, 2H), 3.34 – 3.30 (MeOD), 2.02 – 1.95 (m, 2H), 1.10 – 0.94 (m, 3H).

**GPC** (10 mM LiBr in DMF, PMMA standard) = 57124 Da, *Ð* = 1.25


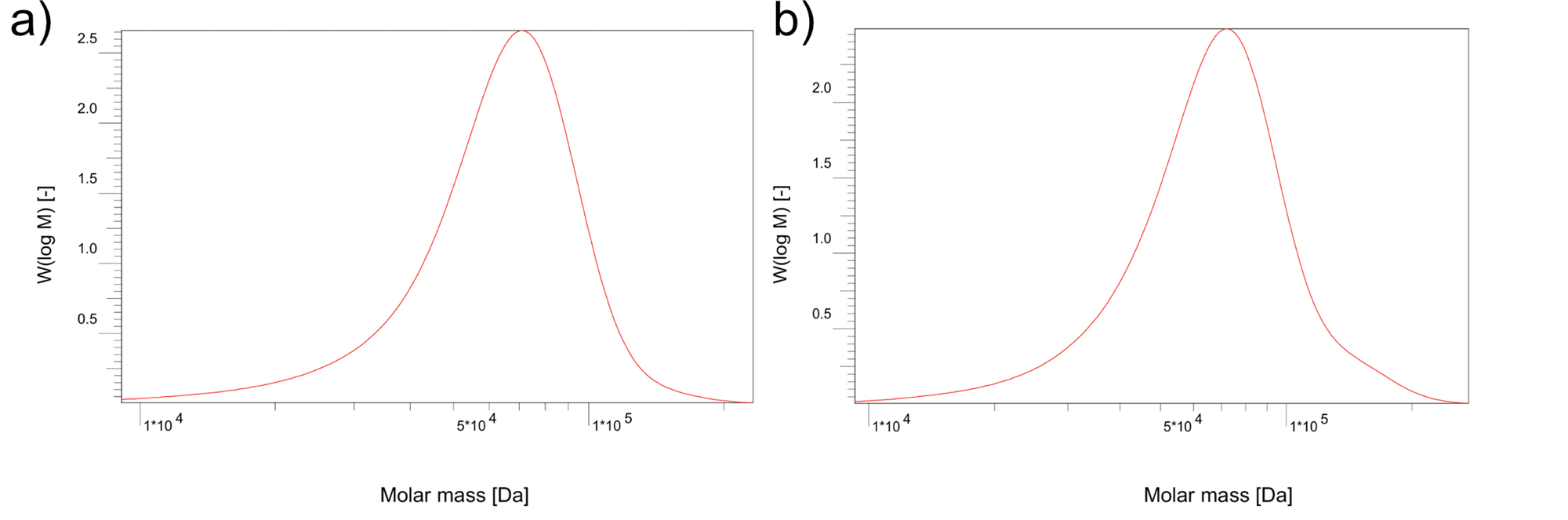


**Figure S10.** GPC chromatogram of **a) P3** homopolymer and **b) P3**-BP block copolymer recorded in DMF (PMMA calibration).

## General Procedure for Sulfation of **P1-2** and **P1-2**-BP

**P1-2** polymers and **P1-2**-BP block copolymers (1 eq) were dissolved in dry DMF. SO_3_∙pyridine was dissolved in dry DMF in a separate flask. Then, the polymer solution was heated to 60 °C before carefully adding SO_3_∙pyridine with a syringe. The mixture was stirred at 60 °C for 2 h and then at room temperature overnight. The reaction was quenched by the addition of 1M NaOH until pH = 11 was reached. DMF was removed by vacuum distillation, yielding a yellow solid. The product was purified by dialysis against salt water (2 M NaCl) for 24 h and in DI water for three more days. After lyophilization, the pure **P1**-**2**-OSO_3_ and **P1**-**2**-OSO_3_-BP products were obtained as a colourless, hygroscopic solid.

## Synthesis and characterization of **P1**-OSO_3_-BP

**P1**-BP = 1.0 g (0.1 mmol, 1 eq), SO_3_ · py = 2.15 g (13.5 mmol, 135 eq.), yield = 50%.


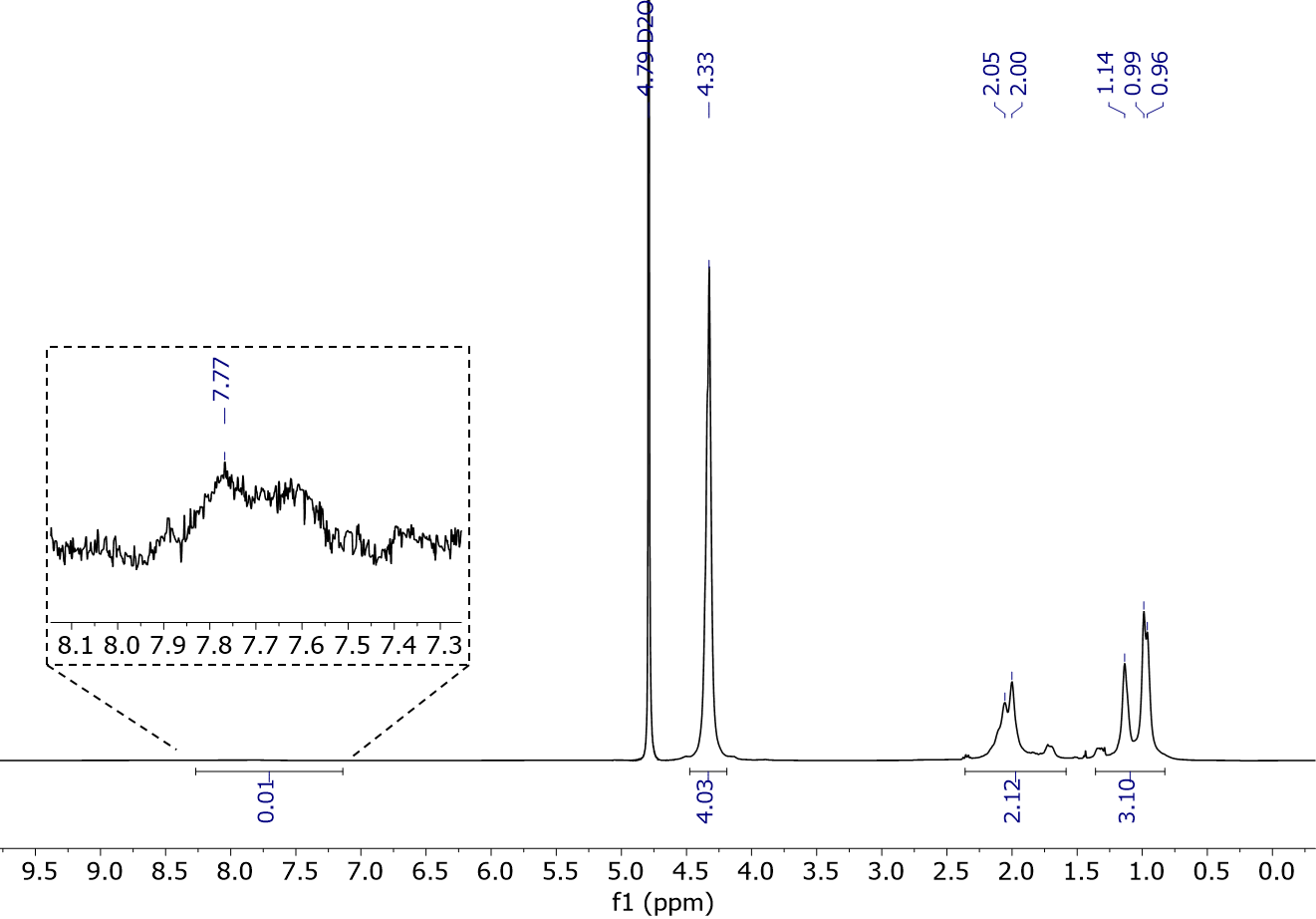


**Figure S11.** ^1^H NMR spectrum of the sulfated (100%) photo-reactive block copolymer **P1**-OSO_3_-BP recorded at 400 MHz in D_2_O.

**^1^H NMR** (400 MHz; D_2_O): δ (ppm) = 7.77 (m), 4.33 (m, 4H), 2.05 – 2.00 (m, 2H), 1.14 – 0.96 (m, 3H).

**GPC** (11.88 mg mL^-1^ Na_2_HPO_4_ in H_2_O (0.08 M), poly (styrene sulfonate) sodium salt standard) (PSS) = 17836 Da, *Ð* = 1.53


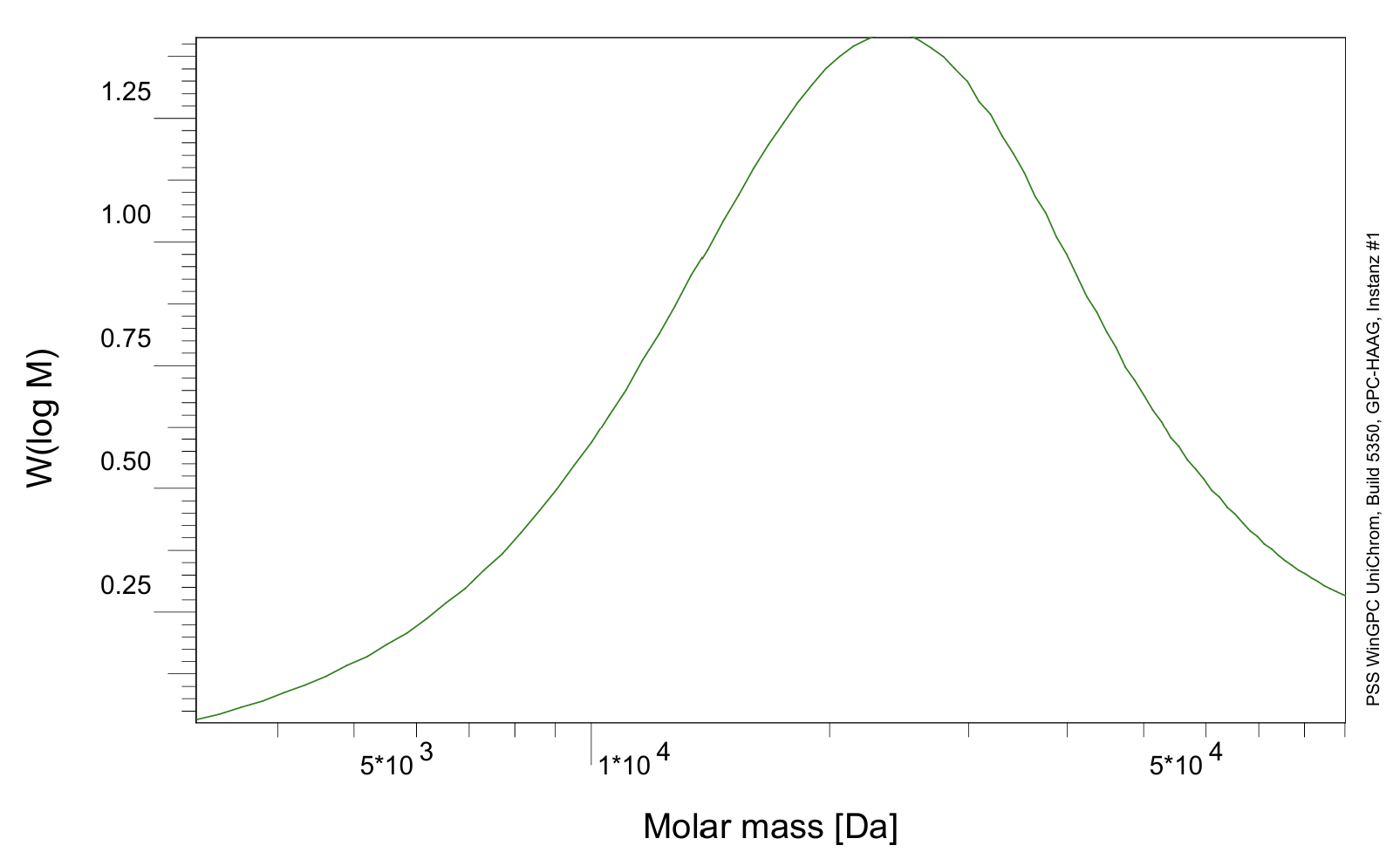


**Figure S12.** GPC chromatogram of **P1**-OSO_3_-BP block copolymer recorded in water (PSS calibration).

## Synthesis and Characterization of **P2**-OSO_3_-BP

**P2**-BP = 1.0 g (21 µmol, 1 eq), SO_3_ · py = 1.39 g (10 mmol, 500 eq.), yield = 41%.

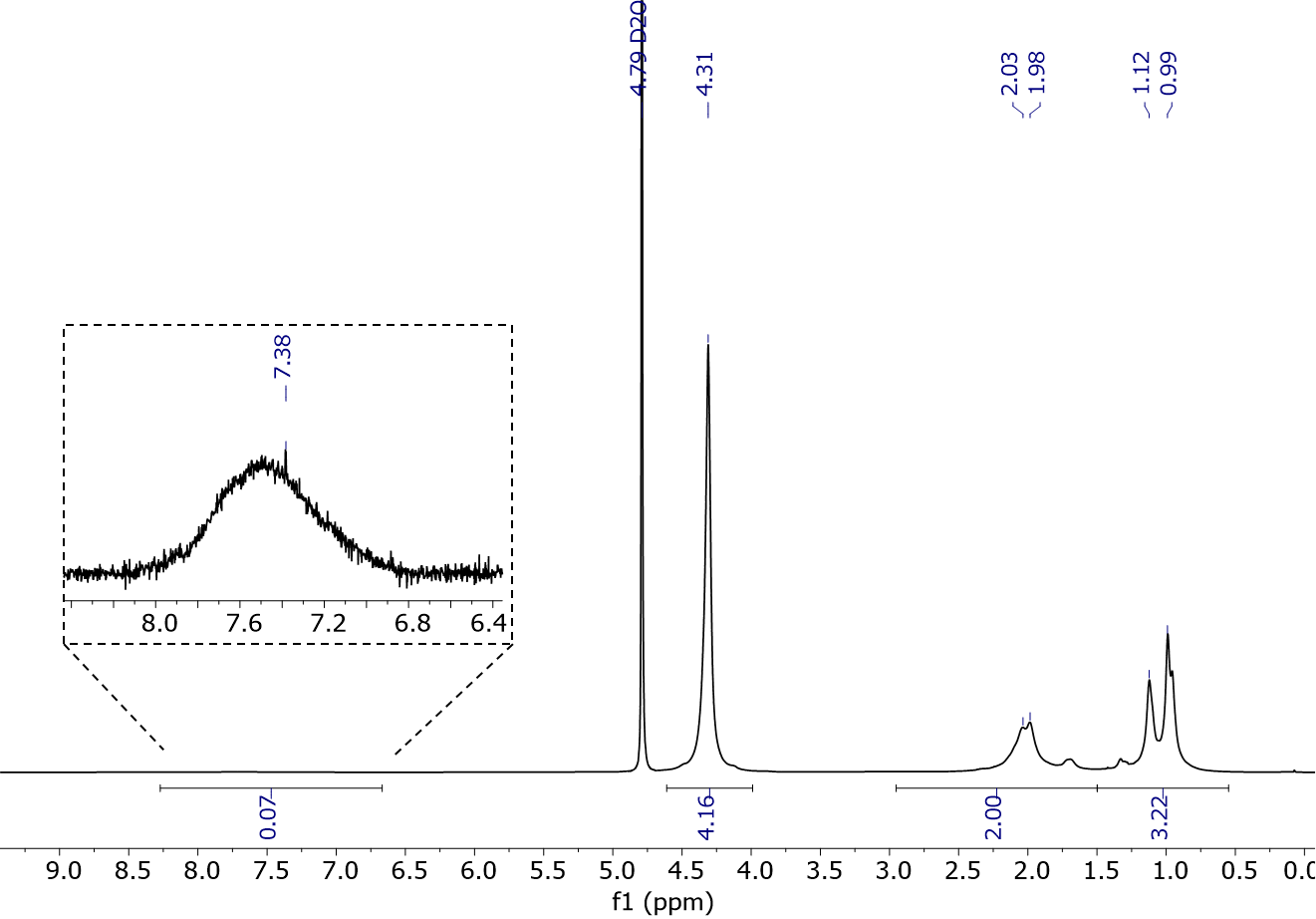


**Figure S13.** ^1^H NMR spectrum of the sulfated (100%) photo-reactive block copolymer **P2**-OSO_3_-BP recorded at 400 MHz in D_2_O.

**^1^H NMR** (400 MHz; D_2_O): δ (ppm) = 7.38 (m), 4.31 (m, 4H), 2.03 – 1.98 (m, 2H), 1.12 – 0.99 (m, 3H).

**GPC** (11.88 mg mL^-1^ Na_2_HPO_4_ in H_2_O (0.08 M), PSS standard) = 108380 Da, *Ð* = 1.98.


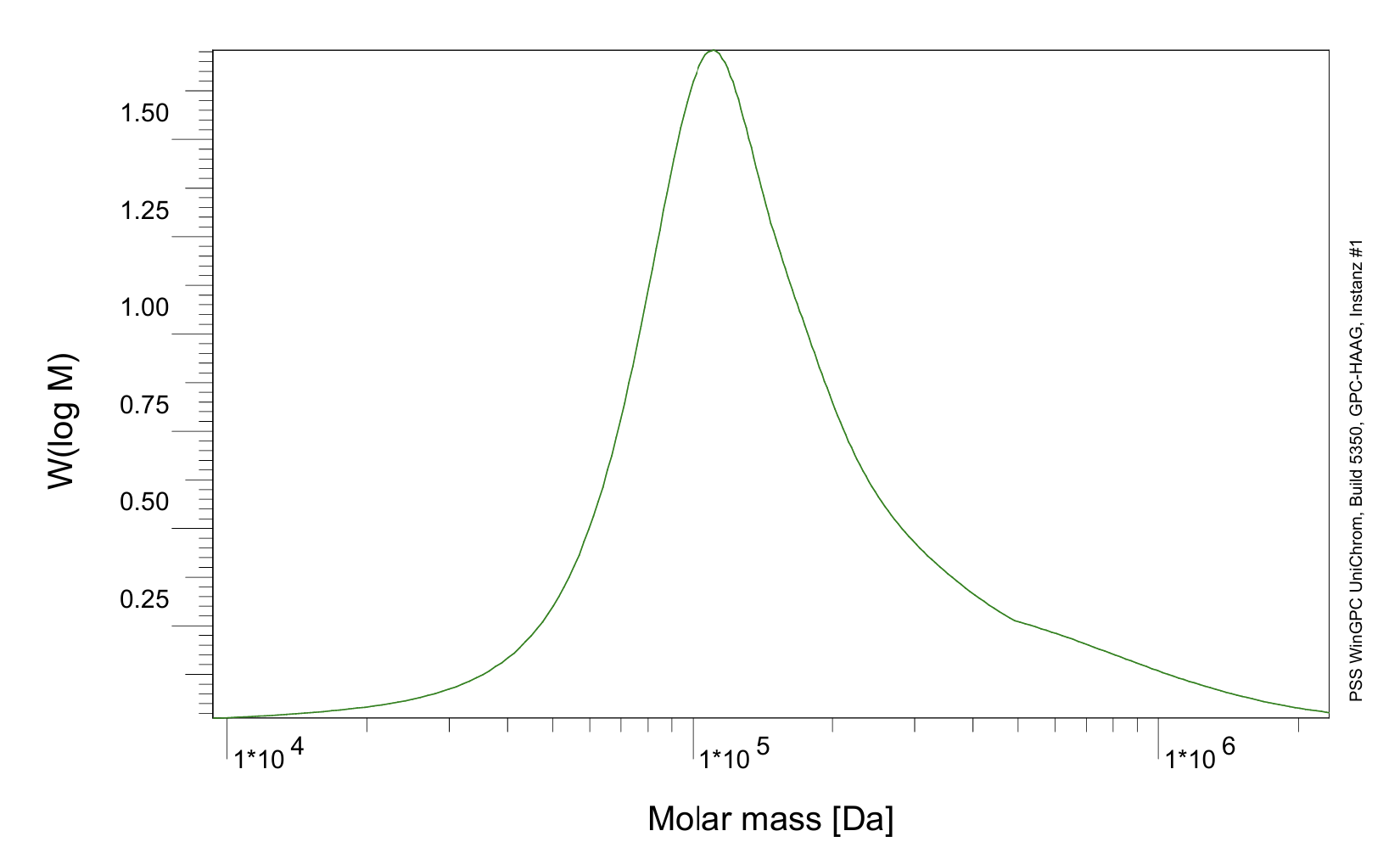


**Figure S14.** GPC chromatogram of **P2**-OSO**3**-BP block copolymer recorded in water (PSS calibration).

# Methods for Surface Modification and Characterization

## Spin Coating of PS Model Substrates

Spin-coating of thin PS films on silicon substrates was performed using a spin coater (WS-650-23) from Laurell Technologies Corporation (North Wales, PA, USA), applying a drop (50 µL) of a PS solution (1 wt-%) in toluene at 3000 rpm for 60 s. Gold sensors for quartz crystal microbalance measurements were coated similarly at 3000 rpm for 60 s using 30 µL of a PS solution (1 wt-%) in toluene. The samples were briefly rinsed with EtOH, subsequently washed with Milli-Q water, and used directly for coating experiments within 1-2 d.

## Surface Functionalization and Characterization

Self-assembled brush coatings on PS surfaces were prepared on PS-coated silicon wafers as model substrates for characterization or PS Petri dishes (Ø 3.5 cm) for cell culture applications. The substrates were statically incubated in 3 mL of the block copolymer solutions (**P3**-BP = 0.25 mg mL^-1^; **P1**-OSO_3_-BP = 0.75 mg mL^-1^; **P2**-OSO_3_-BP = 0.55 mg mL^-1^ ) in aq. EtOH (**P3**-BP) or 0.8 M NaCl (**P1**-OSO_3_-BP, **P2**-OSO_3_-BP) for 90 min in the dark. This polymer brush self-assembly was performed at 40 °C with **P3**-BP or at room temperature with **P1**-OSO_3_-BP and **P2**-OSO_3_-BP. Prior to incubation, the solutions were filtered through a cellulose acetate filter (Labsolute, 0.22 µm). After the supernatant solution was gently discarded from the samples, the surfaces were briefly rinsed with EtOH to remove excess polymer solution (**P3**-BP) or washed with Milli-Q water to remove excess NaCl (**P1**-OSO_3_-BP, **P2**-OSO_3_-BP). After drying under a stream of N_2_, the surfaces were irradiated with UV light in a Beltron UV chamber (λ = 300-700 nm, ~ 10 mW cm^-2^ covered with two borosilicate glass slides (3 mm each) for 16 min to covalently immobilize the physically adsorbed brush layers through their photo-reactive BP-based anchor block. The so-prepared brush structures were extracted in EtOH (**P3**-BP) or Milli-Q water (**P1**-OSO_3_-BP and **P2**-OSO_3_-BP) until the dry layer thickness, measured by spectroscopic ellipsometry (SE), remained constant (~ 1 d). For subsequent contact angle (CA), spectroscopic ellipsometry (SE), quartz crystal microbalance (QCM-D), atomic force microscopy (AFM), and cell culture experiments, the brush coatings were washed with Milli-Q water and dried under a stream of N_2_.

## Polymer Self-Assembly via QCM-D

QCM-D measurements were performed to determine the adhesiveness of PHEMA-based block copolymers towards PS substrates with a one-channel Q-Sense E1 device from LOT-Quantum Design GmbH (Darmstadt, Germany) equipped with a Reglo Digital peristaltic pump from Ismatec (Wertheim, Germany). The software QSoft401 (ver. 2.5.22) was used for data acquisition, and QTools 3 (ver. 3.1.25) from Biolin Scientific AB (Stockholm, Sweden) was used for data analysis. AT-cut crystals with a fundamental resonance frequency of 4.95 MHz were mounted in a standard flow module (Biolin Scientific AB, Stockholm, Sweden) with the polymer-coated side exposed to the flow chamber. The temperature was maintained within ± 0.1 °C for all experiments. The mass sensitivity constant C of the sensor was 17.7 ng cm^-2^ Hz^-1^. All the results in the present study are obtained from the evaluation of the frequency change in the third overtone (*f*_n_= 3).

PS-coated QCM-D gold sensors were used and equilibrated in selective aq. EtOH solutions (EtOH v/v % = 10%) or non-selective EtOH in the QCM-D at 20 °C until the detected frequency changes Δ*f* and dissipation changes Δ*D* were constant. Then, diluted solutions (0.25 mg mL^-1^) of **P3** lacking the BP-based anchor block as well as **P3**-BP in the respective solvents were flown over the sensor under dynamic conditions (0.1 mL min^-1^), followed by a switch to the solvents to remove nondirectionally adsorbed polymer chains until Δ*f* stabilized.

## Cell Culture

HUVECs (passage p. 3-6) and SMCs (p. 3-4) were cultured in their respective VascuLife medium in the presence of 1% penicillin-streptomycin and supplemented with 2 or 5% FBS for HUVECs or SMCs, respectively, for serum-containing culture or without in serum-free culture. Human dermal fibroblasts (HDFs) (p. 3-7) were cultured in DMEM cell culture medium supplemented with 10% FBS and 1% penicillin-streptomycin. All cell types were cultured under standard culture conditions in a humidified atmosphere at 37 °C and 5% CO_2_. Polymer-coated petri dishes for cell culture were disinfected with 70% EtOH for 10 min under the sterile bench and subsequently washed twice with cold DPBS. The sterile dishes were then either used directly after disinfection or stored in sterile conditions in the absence of light for a maximum of 7 days before use. For cell culture in serum-containing media, the polymer-coated dishes and TCPS controls were seeded in 2 mL cell culture medium per dish (HUVECs = 5 x 10^4^ cells cm^-2^, SMCs = 1 x 10^4^ cells cm^-2^; HDF = 3.5 x 10^4^ cells cm^-2^) and cultured (37 °C, 5% CO_2_) for the indicated time. Cells were observed via phase contrast microscopy after 4, 24, and 48 h. For live/dead staining with 50 µM PI and 10 µM FDA in DPBS, cells were incubated in staining solution (2 mL) at 37 °C for 5 min. After aspiration of the staining solution, samples were washed with DPBS and imaged in DPBS using fluorescent mode with appropriate filter sets. In serum-free culture, the polymer-coated dishes and TCPS controls were seeded with cells in 2 mL serum-free cell culture media per dish (1 x 10^4^ cells cm^-2^) and cultured under standard conditions with daily medium change. Cells were observed via phase contrast microscopy at defined timepoints.

## Activated Partial Thromboplastin Time (aPTT)

The aPTT was determined on a STart Max coagulometer. Lyophilized standard human plasma was reconstituted in 1 mL of Milli-Q water by gentle swirling for 15 min. The polymer solutions were prepared in PBS at *c* = 50 mg mL^-1^. Plasma (50 µL), the respective polymer solution (2 µL), and actin FS (50 µL) were added to the measurement cell and incubated at 37°C for 180 s. The measurement was initiated by adding prewarmed 0.025 M CaCl_2_ solution (50 µL). The time required for blood clotting was recorded in seconds, and each test was repeated in triplicate. UFH (0.1 mg mL^-1^) in Milli-Q water was used as a reference. Concentrations given refer to the final concentration of the sample in plasma after addition of the actin solution (1:50).

## Adsorption of VEGF and bFGF

The adsorption of VEGF and bFGF from VascuLife® basal medium was measured with ELISA kits. **P1**-OSO_3_-BP-, **P2**-OSO_3_-BP-, or **P3**-BP-functionalized PS dishes, along with TCPS dishes as controls, were incubated with 1 mL of a 5 ng mL^-1^ of either VEGF- or bFGF-supplemented VascuLife® basal medium. After 2 h incubation at 37 °C in a 5 % CO_2_ incubator, the supernatants were gently mixed by swirling and pipetting, and collected for soluble growth factor quantification via ELISA according to the manufacturer’s instructions with appropriate dilutions to match the detection range of the kits.

# Polymer and Surface Characterization

## Nuclear Magnetic Resonance (NMR) Spectroscopy

^1^H-NMR spectra were recorded using a Joel ECX spectrometer at 400 MHz. The measurements were performed in the specified deuterated solvent (CD_3_OD, D_2_O) at a temperature of 25 °C. The acquired spectra were processed using MestReNova software (ver. 14.2.0), and the chemical shifts are given in δ (ppm) after referencing to the solvent peak as depicted in the NMR spectra.

## Gel Permeation Chromatography (GPC)

GPC measurements of the unsulfated polymers were carried out in DMF with 10 mmol LiBr as eluent at a concentration of 1.5 mg mL^-1^ and a flow rate of 1.0 mL min^−1^ using a customized chromatography system (PSS Polymer Standards Services GmbH, Mainz, Germany). A 5 cm precolumn PSS-SDV (5 μm particle size) coupled with a 30 cm column PSS SDV (5 μm particle size) was used in line with a differential refractometer detector. Calibration was established with PMMA standards from PSS (Mainz, Germany). The temperature of columns was maintained at 50 °C while the differential refractometer detector was set at 35 °C. For sample preparation, 1.5 mg of each polymer was dissolved in 1 mL of the mobile phase (10 mM LiBr in DMF) and filtered through a PTFE (450 μm) filter prior to injection. 50 μL of a filtered sample solution was injected for each measurement. GPC measurements of the sulfated block copolymers were carried out in H_2_O with 11.88 mg mL^-1^ Na_2_HPO_4_ as eluent at a concentration of 3 mg mL^-1^ and a flow rate of 1.0 mL min^−1^ with an Agilent 1100 instrument. A PSS MCX precolumn, along with a second PSS MCX 100000 Å and particle size of 10 µm, was used in line with a differential refractometer detector. Calibration was established with sodium poly(styrene sulfonate). GPC data were processed with PSS Win-GPC software.

## Combustion Analysis

The elemental composition of S, C, and H was analyzed by using an elemental analyzer (Vario EL III, Elementar Analysensysteme GmbH, Langenselbold, Germany). The degree of sulfation was calculated as follows:

The molecular formula of the HEMA r.u. of the unsulfated sample is (C_6_H_10_O_3_)*_n_* and (C_6_H_9_O_6_SNa)*_n_* for the sulfated sample, with *n* being the number of repeating units of the respective r.u.s based on the degree of sulfation calculated by ^1^H NMR analysis. By multiplying the molecular weight of the repeating unit times the number of repeating units *n* in **P1**-**2**-OSO_3_ – as determined from the ^1^H NMR spectra – and adding the molecular weight of the RAFT chain end-groups (C_13_H_13_NO_2_S_2_), the total atomic masses for the C-, S-, and H-atoms were calculated according to the following equation:

*M_X_* [g mol^-1^] = [occurrence*_X_* · atomic mass*_X_* _·_ *n*] **_P1_**_-_**_2_**_-OSO3_ + [occurrence*_X_* · atomic mass*_X_*]_end-groups_

with *x* corresponding to C, S, or H and occurrence corresponding to the numerical appearance of the respective atom in the repeating unit. The elemental mass-% fraction of each element was then calculated by relating the so-calculated relative masses to the overall molecular weight of the sulphated polymer. Theoretical (X^theo^) and experimental mass fractions of the individual atoms (X^CHS^) are summarized in Table S1 and compared to those determined by ^1^H NMR analysis (X^NMR^).

## Spectroscopic Ellipsometry (SE)

For the characterization of PS films, the dry layer thickness, as well as the refractive index, were measured by SE at an incident angle of 70° and wavelengths from 370 to 1070 nm with a SENpro spectroscopic ellipsometer from SENTECH Instruments GmbH (Berlin, Germany) and calculated as an average value of five different spots on the surface and further used as fixed values for the subsequent modelling of the brush layers. The dry thickness of the brush layers was determined similarly as average of five different spots on the surface by fitting a model consisting of a silicon dioxide layer, a PS layer with fixed parameters (dry thickness = 83.86 ± 2.48 nm, refractive index = 1.651 ± 0.004), and a Cauchy layer – the layer to be determined – with a fixed refractive index *n* = 1.51 and air as the surrounding medium. At least four independent substrates (n = 4) were measured to test for reproducibility.

- 1. *Water Contact Angle (CA)*

The wettability of the brush coatings was determined by static CA measurements with an OCA contact angle system from DataPhysics Instruments GmbH (Filderstadt, Germany) and fitted with the software package SCA202 (ver. 3.12.11) using the sessile drop configuration. CAs of the immobilized hydrogels were determined after extraction of non-crosslinked chains at 20 °C. Therefore, a drop of Milli-Q water (2 µL) was placed onto the surface, and CAs were determined right after deposition with the Young-Laplace model. For each substrate, CAs were measured on five different spots to test for sample homogeneity and at least four independent substrates (n = 4) to test for reproducibility.

- 1. *Dynamic Light Scattering (DLS)*

Dynamic light scattering analysis was performed using a Zetasizer Ultra (Malvern Panalytical Ltd., Malvern, United Kingdom) in backscattering mode (detection angle = 173°) at the specified temperatures. Measurements were performed in two consecutive triplicate using quartz cuvettes supplied by Hellma Analytics GmbH (Müllheim, Germany) with a volume of 2 mL, and each sample was thermally equilibrated for at least 5 min prior to measurements. Data evaluation was performed using the software ZS Xplorer (Malvern Panalytical Ltd., Malvern, United Kingdom).

- 1. *Optical Microscopy*

Microscopic images of HUVECs, SMCs, and HDFs were taken on a Zeiss Observer Z1 from Carl Zeiss Microscopy GmbH (Jena, Germany) and processed with the software Zen 3.5 ver. 3.5.093.00001. Cell surface coverage was analyzed with a custom Fiji Macro.[3]

- 1. *Atomic Force Microscopy (AFM)*

The morphology of the polymer brush coatings on PS-functionalized silicon wafers was analysed by AFM. A NanoWizard IV AFM (JPK Instruments, Berlin, Germany) mounted on top of an LSM800 confocal microscope (Carl Zeiss, Jena, Germany) was used for imaging the surface of the samples. The AFM head was equipped with a 15 µm z-range linearized piezoelectric scanner and an infrared laser. Imaging of the samples was performed in air in tapping mode with PointProbePlus® Non-Contact – Long Cantilever – Reflex Coating (PPP-NCLR) silicon sensors with a tip radius of approximately 7 nm, nominal resonant frequency of 170 kHz, and spring constant of 30 N/m. Imaging parameters were adjusted to minimize the force applied to the surface. Scanning speed was optimized to 0.5 – 0.7 Hz, and acquisition points were 256 x 256. Images were analysed with the JPK image processing software v.6.4.21 (JPK Instruments, Berlin, Germany).

- 1. *X-Ray Photoelectron Spectroscopy (XPS)*

XPS experiments were performed with an EnviroESCA spectrometer (SPECS Surface Nano Analysis GmbH, Berlin, Germany), equipped with a monochromatic Al Kα X-ray source (Excitation Energy = 1486.71 eV) and a PHOIBOS 150 electron energy. All spectra were measured in normal emission, and a source-to-analyzer angle of 55° was used. The spectra were acquired in fixed analyzer transmission (FAT) mode. The binding energy scale of the instrument was calibrated, following a technical procedure provided by SPECS Surface Nano Analysis GmbH (calibration was performed according to ISO 15472). For quantification, the survey spectra were acquired at ultra-high vacuum conditions (p < 1 x 10^-5^ mbar) with a pass energy of 100 eV, and the spectra were quantified utilizing the empirical sensitivity factors that were provided by SPECS Surface Nano Analysis GmbH (the sensitivity factors were corrected with the transmission function of the spectrometer).

## 5.0 Estimation of scVEGF surface density from reference [3][4]

Based on the information provided in ref [3], the achieved surface density of engineered single-chain VEGF (scVEGF; composed of two GCC-linked 3-112 amino acid fragments of VEGF_121_) bioconjugated to fibronectin (FN) on a 96-well plate (0.32 cm^2^) can be estimated. The scVEGF equipped a terminal Cys-tag (~25 kDa) was decorated on monomeric fibronectin (FN, ~ 250 kDa ) at an average of ~5-6 scVEGF per FN. The resulting FN-scVEGF was estimated to have a molecular weight of ~390 kDa. Surface coatings have been produced from 0.01-1 pmol FN-scVEGF per well with an immobilization efficiency of ~13-27% of the initially added protein. Based on the dose-dependent cell response observed with *n* = 0.01 - 0.2 pmol FN-scVEGF per well, the scVEGF surface density can be estimated:

scVEGF cm^-2^ = (*n* (FN-scVEGF) x 5.5 x 25000 g/mol x 0.2) / 0.32 cm^2^

By doing so, we estimated surface densities of ~ 0.86 ng cm^-2^ up to 17.2 ng cm^-2^.

# Supplementary Figures

**Table S1.** Theoretical and experimental atomic percentages as determined by elemental analysis and ^1^H NMR spectroscopy.

| Polymer | C^theo^  [%] | C^CHS^  [%] | C^NMR^  [%] | H^theo^  [%] | H^CHS^  [%] | H^NMR^  [%] | S^theo^  [%] | S^CHS^  [%] | S^NMR^  [%] |
| --- | --- | --- | --- | --- | --- | --- | --- | --- | --- |
| **P1-**OSO_3_-40 | 42 | 40 | 41 | 5 | 5 | 6 | 8 | 8 | 6 |
| **P1-**OSO_3_-80 | 32 | 34 | 29 | 4 | 4 | 5 | 13 | 14 | 11 |
| **P1-**OSO_3_-100 | 31 | 32 | 28 | 4 | 4 | 4 | 14 | 14 | 12 |
| **P2-**OSO_3_-40 | 41 | 47 | 41 | 5 | 6 | 7 | 5 | 10 | 6 |
| **P2-**OSO_3_-80 | 34 | 38 | 31 | 4 | 5 | 5 | 4 | 14 | 10 |
| **P2-**OSO_3_-100 | 31 | 33 | 27 | 4 | 4 | 4 | 4 | 15 | 12 |
| **P2-**OSO_3_-BP | 33 | 33 | 27 | 4 | 4 | 4 | 13 | 13 | 11 |
| **P2-**OSO_3_-BP (UV) | 33 | 33 | 29 | 4 | 4 | 4 | 13 | 13 | 13 |

**
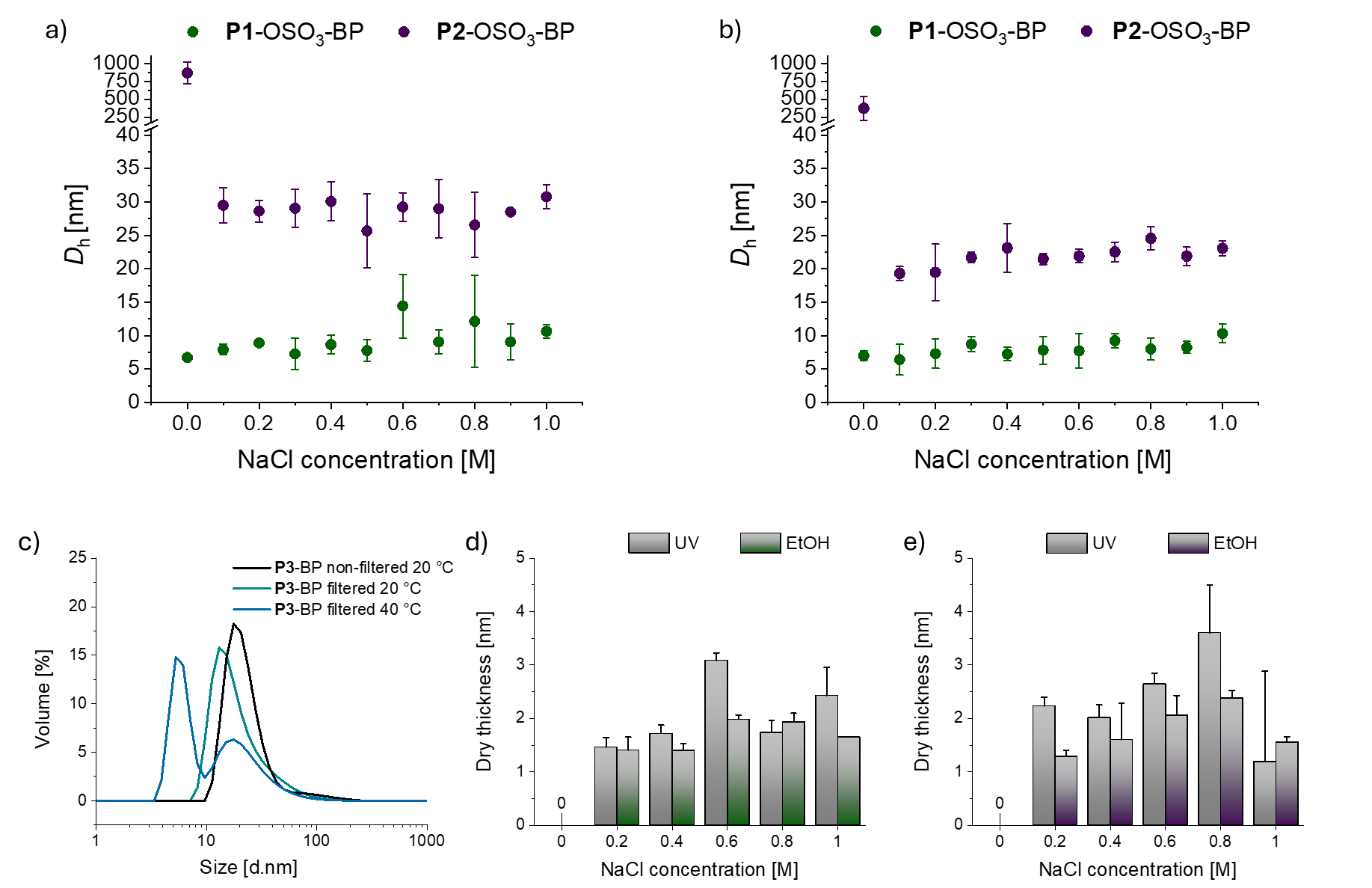
**

**Figure S15.** Mean hydrodynamic diameter *D*_h_ by volume of **P1**-OSO_3_-BP (green) (0.75 mg mL^-1^, 2.6 x 10^-5^ M) and **P2**-OSO_3_-BP (purple) (0.55 mg mL^-1^, 5.3 x 10^-6^ M) in Milli-Q water and NaCl solutions **a)** before and **b)** after filtration through a 0.2 µm filter. **c)** Representative DLS curves of **P3**-BP in aq. EtOH (250 µg mL^-1^) at 20 and 40 °C before and after filtration through a 0.2 µm cellulose acetate filter. Dry layer thickness as measured via SE after UV immobilization (grey bars) and water extraction (coloured bars) of **d)** **P1**-OSO_3_-BP and **e) P2**-OSO_3_-BP brushes on PS-coated silicon wafer substrates. (n = 3)

DLS measurements of **P1**-OSO_3_-BP and **P2**-OSO_3_-BP with increasing salt solution before and after syringe filtration (**Figure S15 a,b**) indicated mainly singularized chains for **P1**-OSO_3_-BP in the presence and absence of salt and stronger singularization of **P2**-OSO_3_-BP after filtration in the presence of salt. Furthermore, 20 nm aggregates of **P3**-BP with narrow dispersity at 20 °C (Figure S6c) were detected; upon heating to 40 °C and filtration through a 0.2 µm syringe filter, two smaller populations appeared. These likely reflect disassembly of the hydrophobicity-driven aggregates into single chains (6 nm) and looser aggregates (13 nm) at higher temperatures, overcoming weak Van-der Waals and hydrogen bonding interactions of the BP blocks. Filtration shear stress alone at 20 °C did not significantly alter the hydrodynamic diameter.

Thickness analysis of **P1**-OSO_3_-BP and **P2**-OSO_3_-BP demonstrated that the efficient self-assembly of polyelectrolyte brushes only occurs from saline solutions and not from pure water. This is attributed to the BP-induced micellar conformation of **P1**-**2**-OSO_3_-BP block copolymers in the absence of salt, that hinders the exposure of the BP-based anchor toward the PS substrate. A loss in brush layer thickness ≤ 30% after UV irradiation and extraction strongly suggests the correct orientation of the self-assembled polymer chains.

**
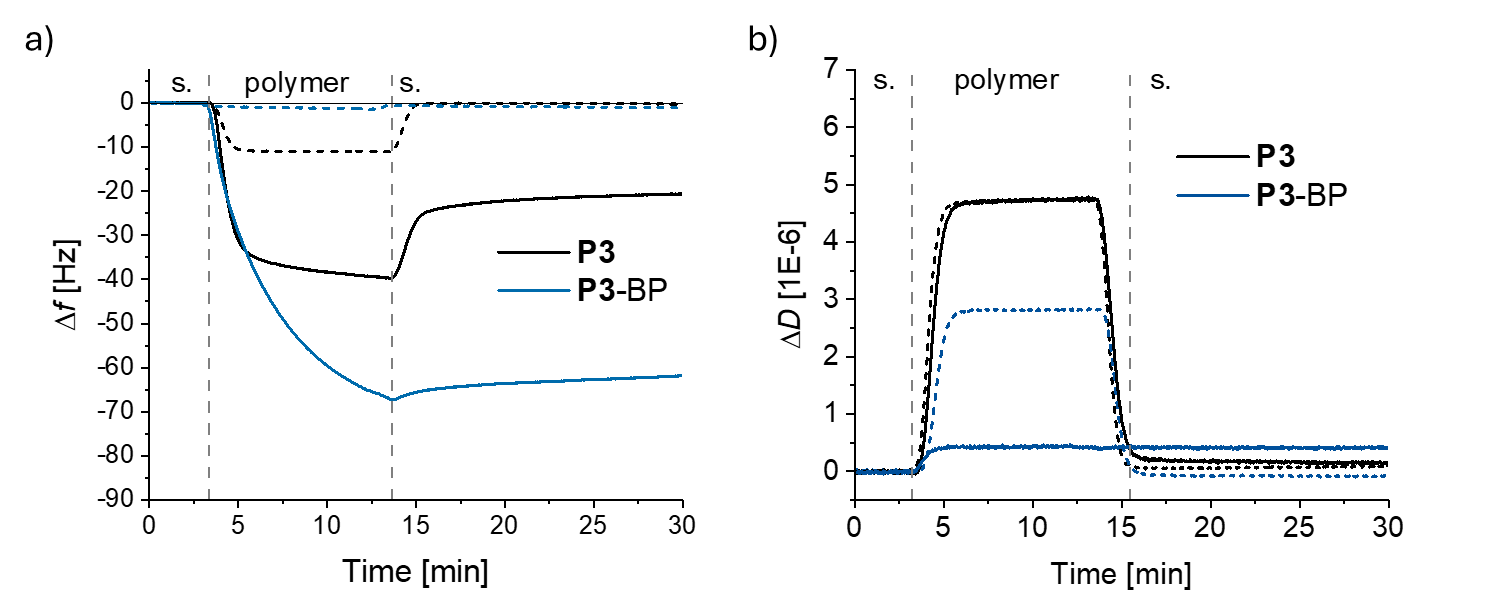
**

**Figure S16.** Interaction of PHEMA-based homo- and block copolymers with PS-surfaces assessed via QCM-D measurements from selective (10 v/v% EtOH in water, solid) and non-selective solvents (EtOH, dashed) at 20 °C. Representative **a)** frequency shift (Δ*f* ) and **b)** dissipation shifts (Δ*D*) for the adsorption of block copolymer **P3**-BP (blue) and homopolymer **P3** lacking the BP block (black) on PS-coated gold sensor chips, indicating a specific interaction of the BP anchor block with the substrate surface under selective solvent conditions. Vertical dashed lines indicate medium changes from solvent (s.) to polymer solution and vice versa. (n = 3)


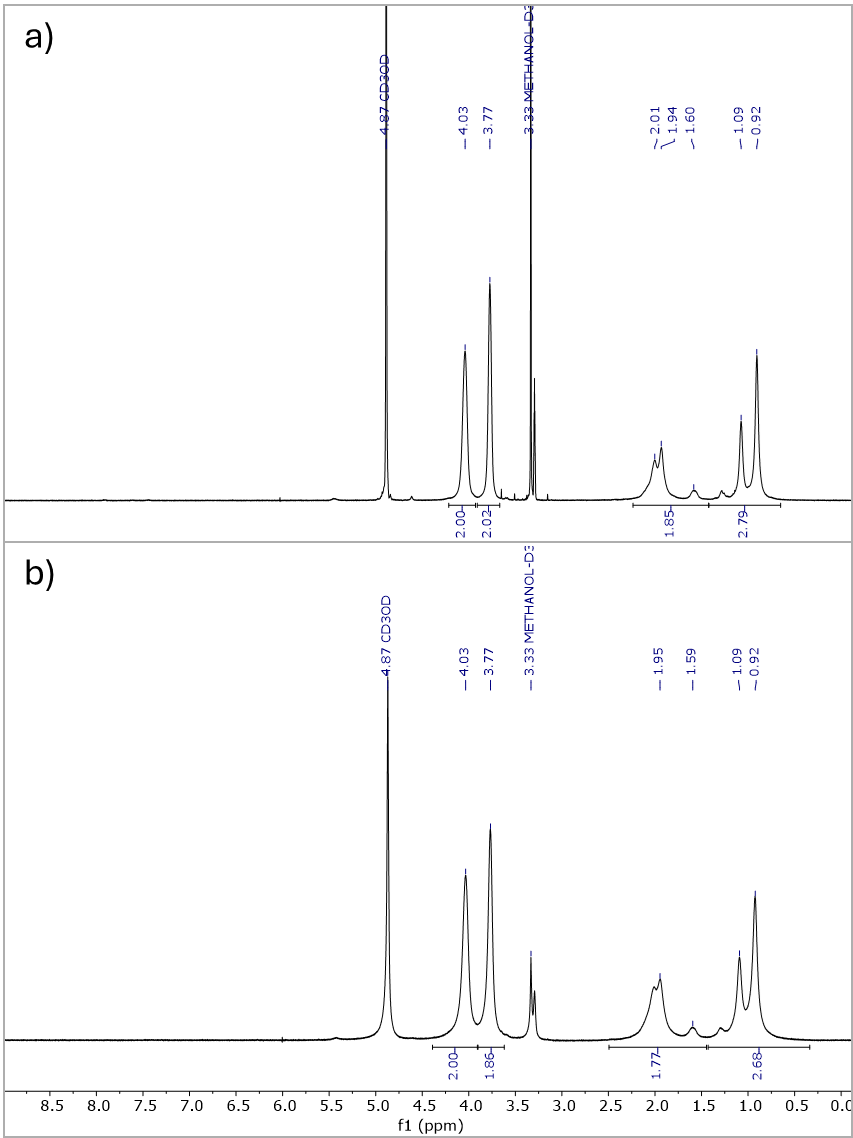


**Figure S17.** ^1^H-NMR spectrum of **P3** **a)** before and **b)** after 16 min UV-irradiation recorded at 400 MHz in CD_3_OD.

**Calculation of the brush grafting density (*GD*):**

$GD= \frac{\delta d_{h}N_{A}}{M_{n}}$ (eq. S1)

where *δ* corresponds to the bulk density of PHEMA (*δ=*1.07 g cm^-3^),[5] *d*_h_ is the dry thickness of the immobilized brushes after extraction, *N*_A_ is Avogadro’s number, and *M*_n_= 29000 g mol^-1^ for **P1**-OSO_3_-BP, 117000 g mol^-1^ for **P2**-OSO_3_-BP, and 37000 g mol^-1^ for **P3**-BP.

The calculated brush grafting density (GD) was used to estimate the average distance between grafting points (*L*) assuming a square-lattice model according to

$L\approx\left( GD \right)^{-0.5}$ (eq. S2)

in order to estimate the degree of chain overlap based on polymer brush theory.[6] Chain overlap occurs when the interchain distance *L* becomes comparable to twice the polymer´s radius of gyration *R*_g_. Here, *R*_g_ was estimated from the number of repeating units r.u. of the polymer by using *R*_g_ = (0.181 r.u.)^0.5^ nm.[5] The degree of chain overlap, expressed as *L*/2*R*_g_, distinguishes between conformational regimes: values greater than unity (*L*/2*R*_g_ > 1) correspond to isolated polymer chains in the mushroom or pancake regime, whereas values below unity (*L*/2*R*_g_ < 1) indicate chain overlap and the onset of brush-like behaviour, where steric and excluded volume interactions, together with electrostatic repulsion between charged sulfate groups, force chains to extend away from the surface.


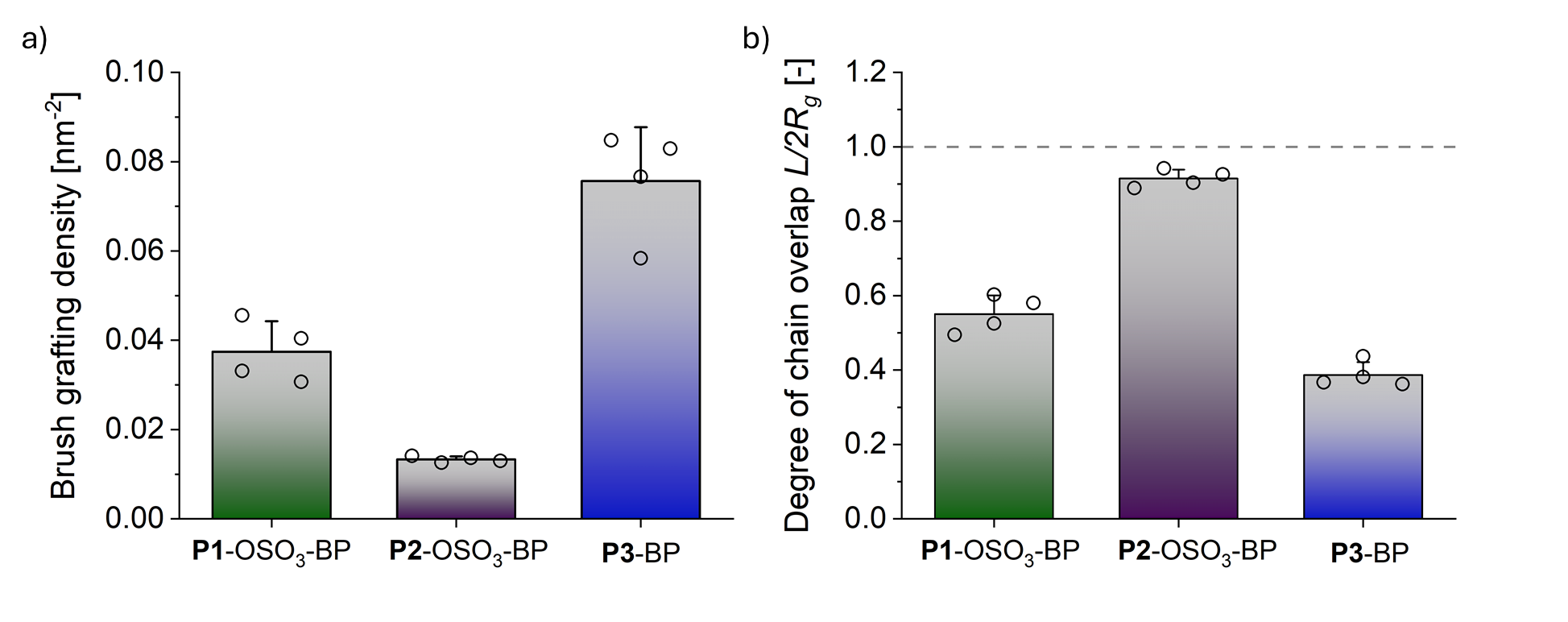


**Figure S18**. **a)** Brush grafting density and **b)** corresponding degree of chain overlap, expressed as *L*/2*R*_g_, calculated at the respective grafting densities for **P1**-OSO_3_-BP (green), **P2**-OSO_3_-BP (purple), and **P3**-BP (blue). Data are plotted for each measured replicate with their mean value (bars) and SD. Grey dotted line represents the transition from mushroom or pancake conformations (*L*/2*R*_g_ > 1) to the brush regime (*L*/2*R*_g_ < 1) characterized by a significant chain overlap.

**
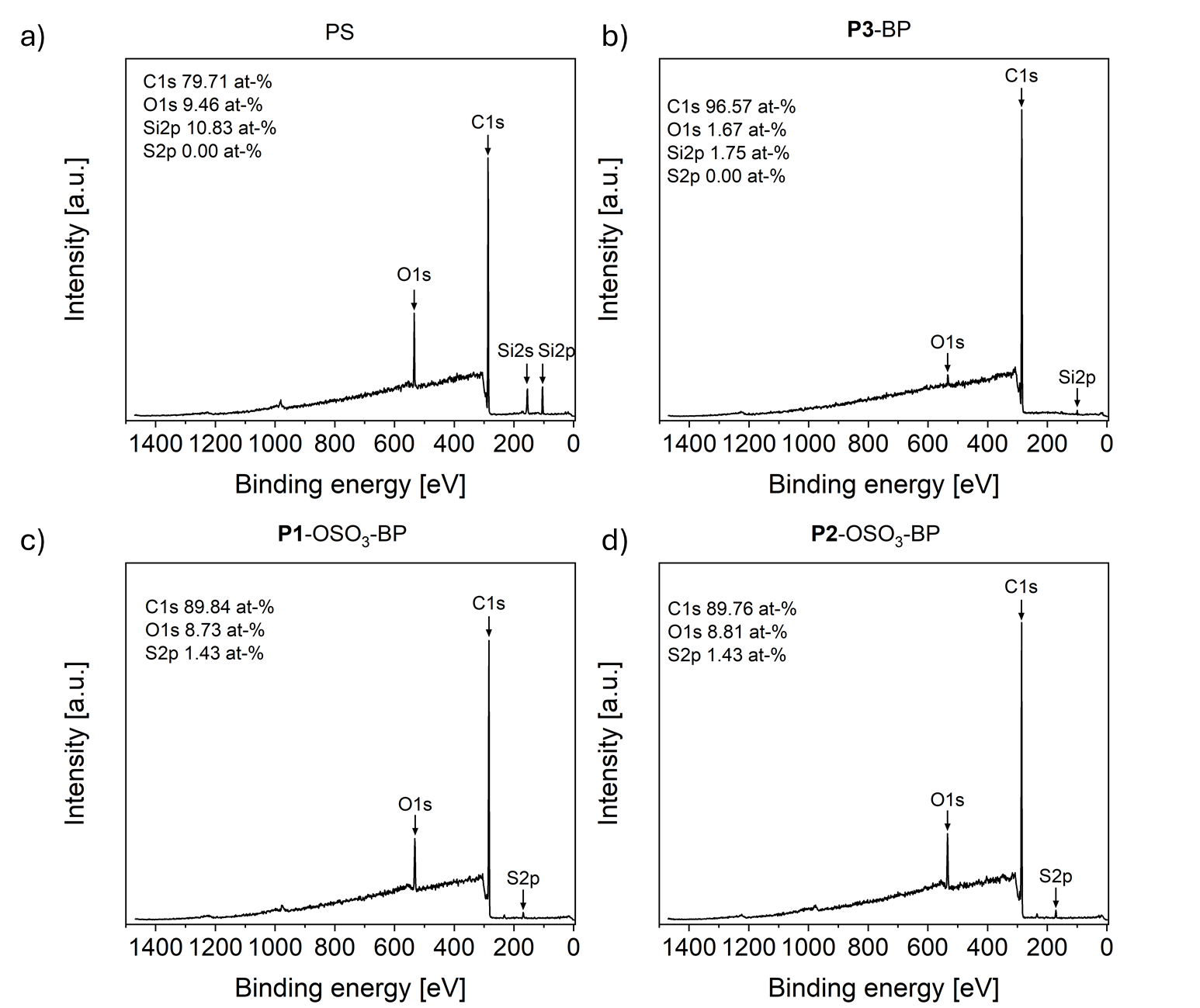
**

**Figure S19.** XPS survey spectra of **a)** PS basal layer, **b)** non-sulfate control **P3**-BP, **c) P1**-OSO_3_-BP, and **d) P2**-OSO_3_-BP brushes, including peak assignment.


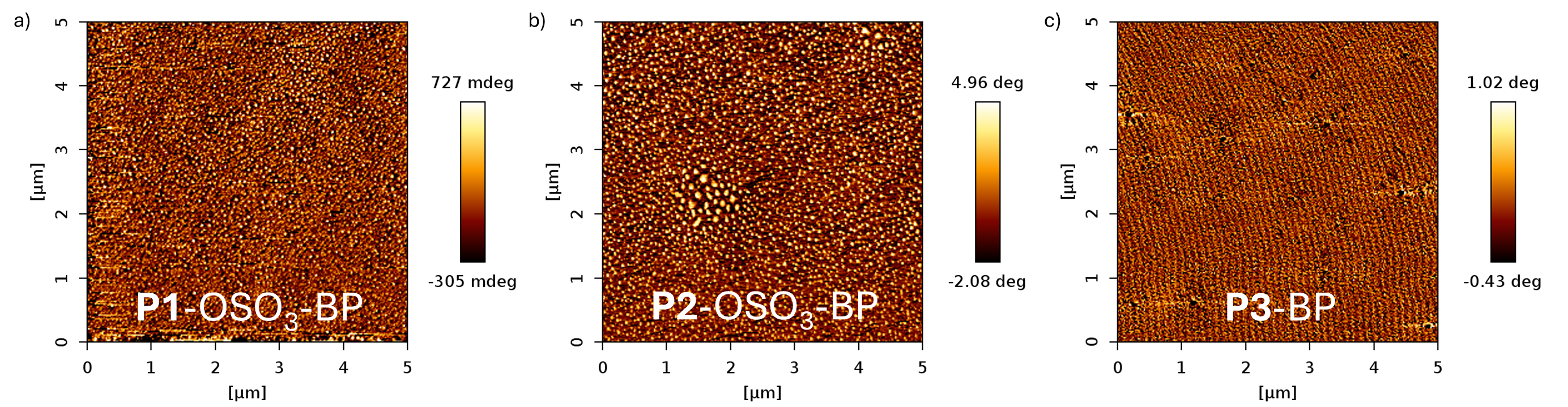


**Figure S20.** Representative AFM phase images of self-assembled and UV-immobilized brushes of **a)** **P1**-OSO_3_-BP, **b) P2**-OSO_3_-BP, and **c) P3**-BP on PS-coated Si wafers in air.


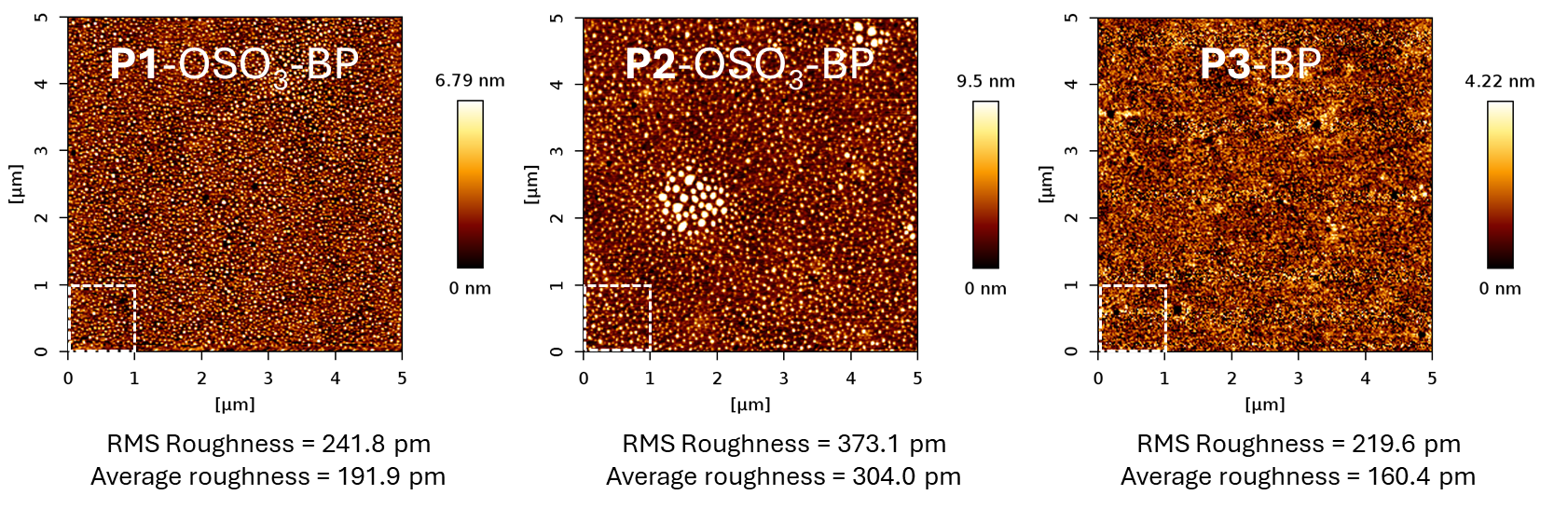


**Figure S21.** Representative AFM topography and the respective roughness parameters averaged over a 1 µm^2^ area (white dotted box).


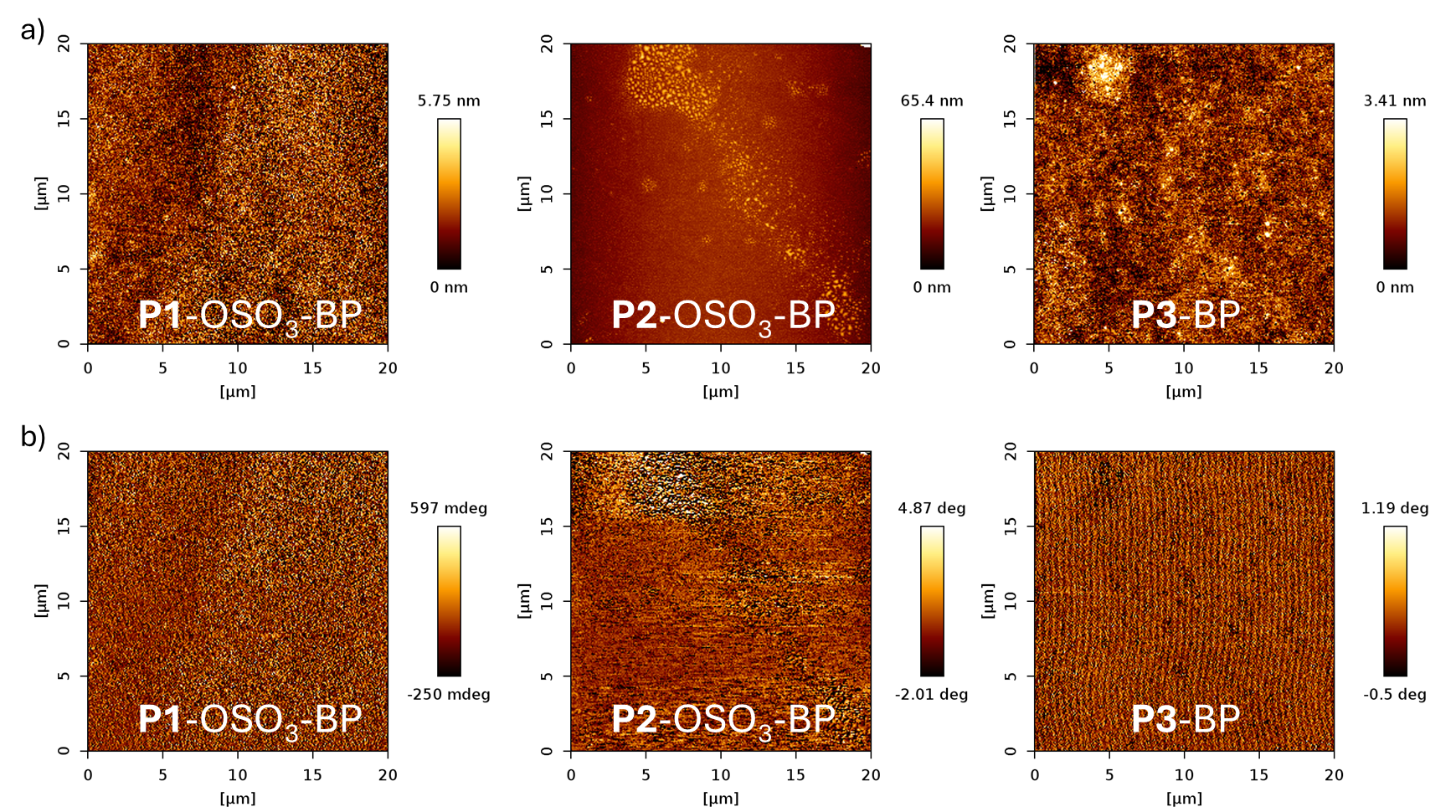


**Figure S22.** Representative AFM **a)** height and **b)** phase images of self-assembled and UV-immobilized brushes of **P1**-OSO_3_-BP, **P2**-OSO_3_-BP, and **P3**-BP, on PS-coated Si wafers in air.


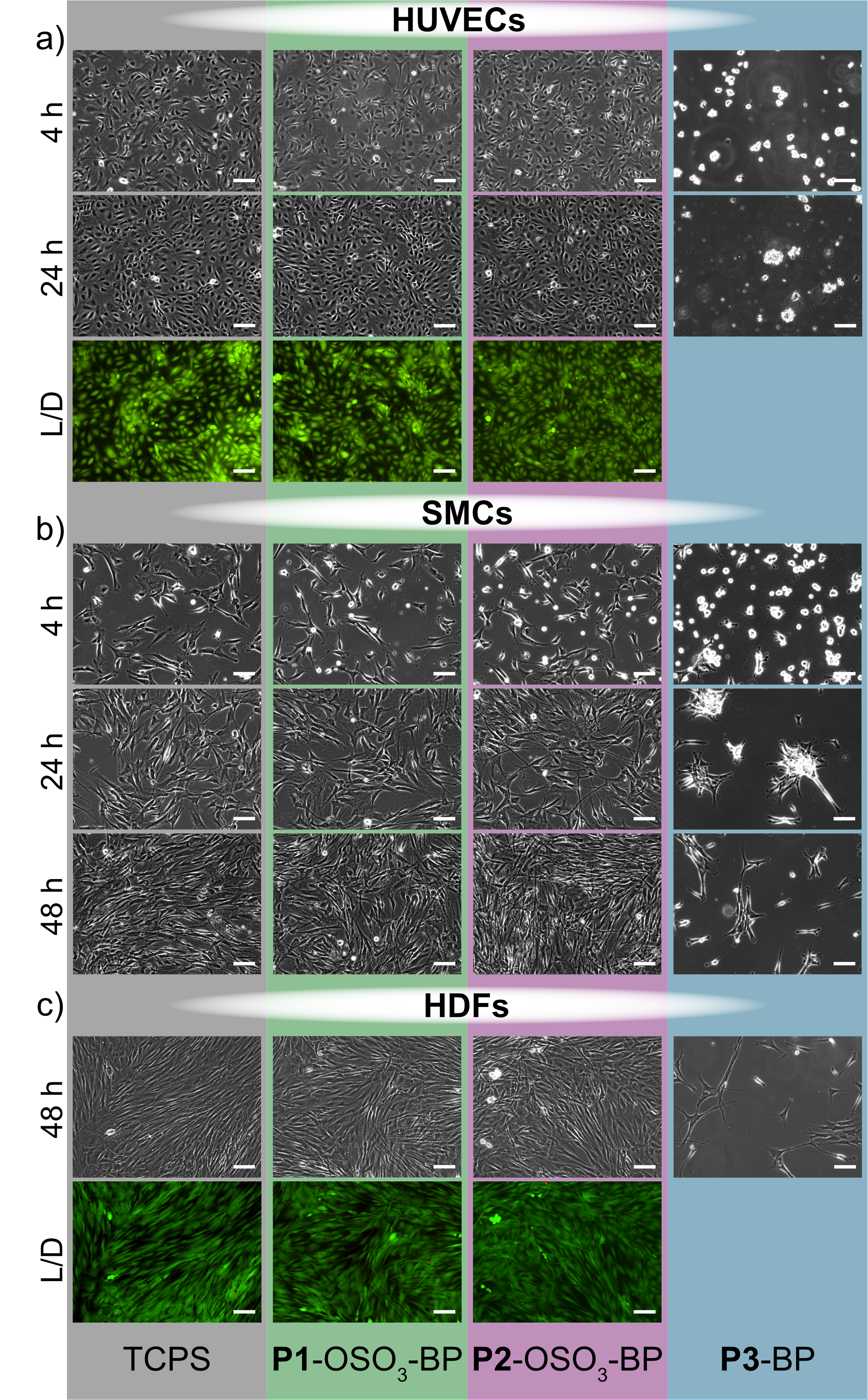


**Figure S23.** Representative phase contrast and fluorescence images of **a)** HUVECs cultured for 24 h, as well as **b)** SMCs and **c)** HDFs cultured for 48 h in serum-containing medium on **P1**-OSO_3_-BP, **P2**-OSO_3_-BP, and TCPS control after live/dead (L/D) staining with PI (red/D) and FDA (green/L) (merged channels). HUVECs = 5 x 10^4^ cells cm^-2^; SMCs = 1 x 10^4^; HDF = 3.5 x 10^4^ cells cm^-2^. Scale bar = 100 µm.

**
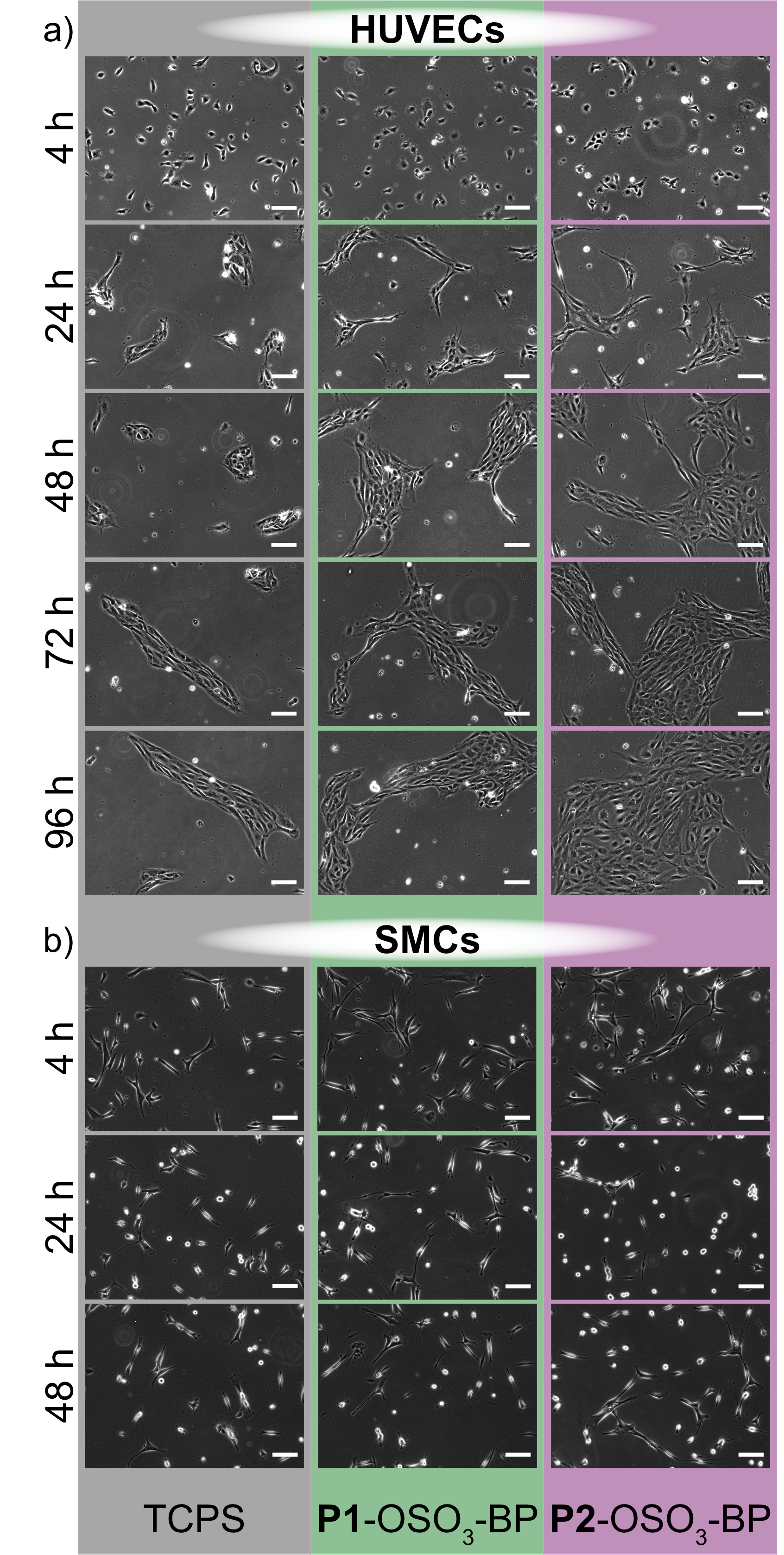
**

**Figure S24.** Representative phase contrast images of **a)** HUVECs after 4, 24, 48, 72, and 96 h and **b)** SMCs after 4, 24, and 48 h after seeding on **P1**-OSO_3_-BP, **P2**-OSO_3_-BP, and TCPS controls in serum-free medium. Seeding density: 1 x 10^4^ cells cm^-2^. Scale bar = 100 µm. (n = 3)

**
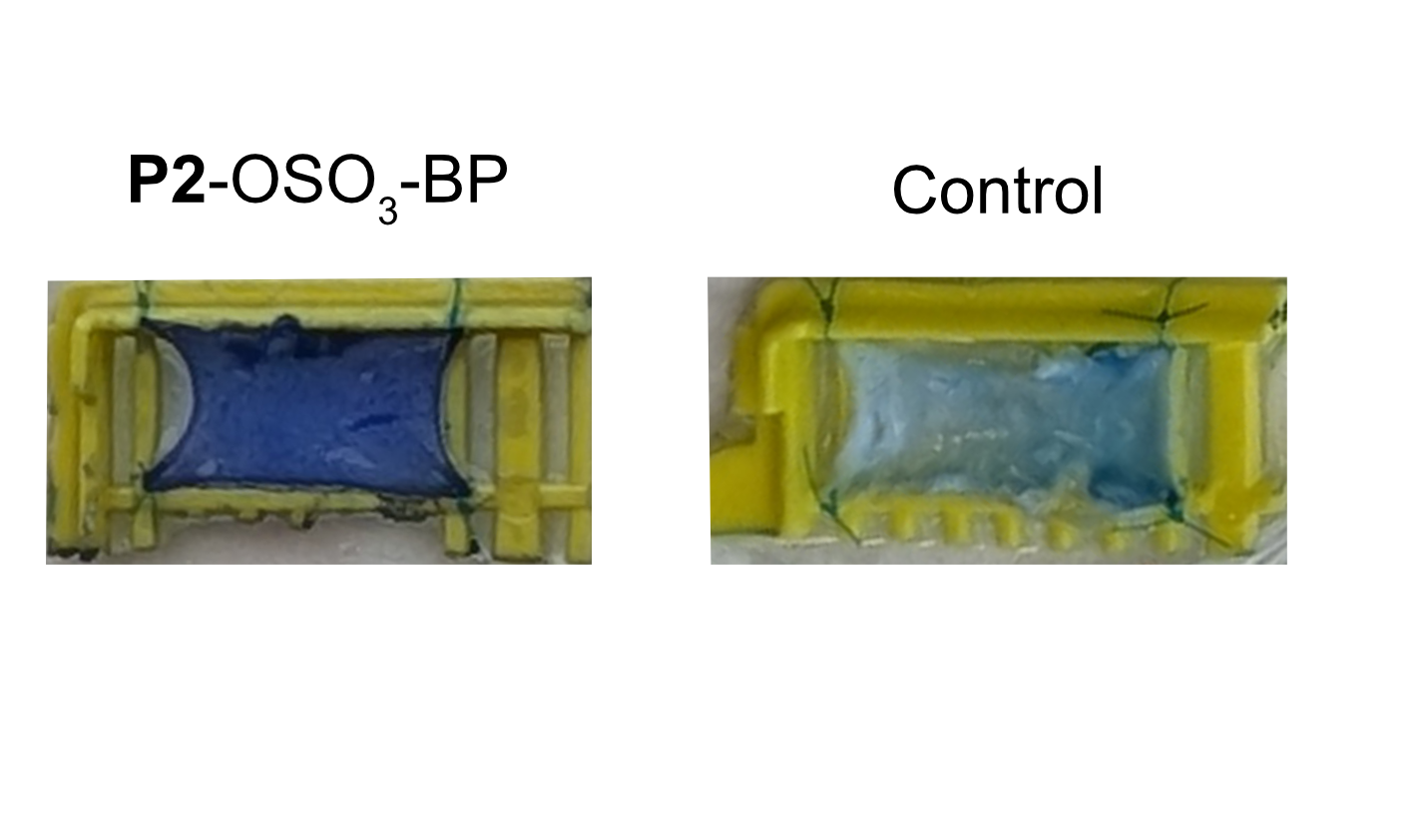
**

**Figure S25.** Macroscopic photographs of methylene blue-stained decellularized extracellular matrix of a rat aorta cut and fixed in the yellow plastic frame before (right side) and after (left side) functionalization with **P2**-OSO_3_-BP.

**REFERENCES**

[1]Stöbener, D. D.; Uckert, M.; Cuellar-Camacho, J. L.; Hoppensack, A.; Weinhart, M. Ultrathin Poly(Glycidyl Ether) Coatings on Polystyrene for Temperature-Triggered Human Dermal Fibroblast Sheet Fabrication. *ACS Biomater. Sci. Eng.* **2017**, *3* (9), 2155-2165. DOI: 10.1021/acsbiomaterials.7b00270.

[2]Schweigerdt, A.; Stöbener, D. D.; Scholz, J.; Schäfer, A.; Weinhart, M. Thermoresponsive Brush Coatings for Cell Sheet Engineering with Low Protein Adsorption above the Polymers’ Phase Transition Temperature. *ACS Appl. Bio Mater.* **2024**, *7* (11), 7544-7555. DOI: 10.1021/acsabm.4c01127.

[3]Kerkhoff, Y.; Cosimi, A.; Ludwig, K.; & Weinhart, M. Automatic Quantification of Cell Surface Coverage of Mono and Co-Cultures Based on Optical Microscopy and Small Neural Networks with Fiji. Zenodo 2025. DOI: 10.5281/zenodo.17581056

[4]Backer, M. V.; Patel, V.; Jehning, B. T.; Claffey, K. P.; Backer, J. M. Surface Immobilization of Active Vascular Endothelial Growth Factor Via a Cysteine-Containing Tag. *Biomaterials* **2006**, *27* (31), 5452-5458. DOI: 10.1016/j.biomaterials.2006.06.025.

[5]Deng, J.; Ren, T.; Zhu, J.; Mao, Z.; Gao, C. Adsorption of Plasma Proteins and Fibronectin on Poly(Hydroxylethyl Methacrylate) Brushes of Different Thickness and Their Relationship with Adhesion and Migration of Vascular Smooth Muscle Cells. *Regen. Biomater.* **2014**, *1* (1), 17-25. DOI: 10.1093/rb/rbu008 (acccessed 4/10/2026).

[6]de Gennes, P. G. Conformations of Polymers Attached to an Interface. *Macromolecules* **1980**, *13* (5), 1069-1075. DOI: 10.1021/ma60077a009.
